# Digital Graphic Follow-up Tool (Rehabkompassen) for Identifying Rehabilitation Needs Among People After Stroke: Randomized Clinical Feasibility Study

**DOI:** 10.2196/38704

**Published:** 2022-07-29

**Authors:** Xiaolei Hu, Karolina Jonzén, Olof A Lindahl, Marcus Karlsson, Fredrik Norström, Erik Lundström, Katharina Stibrant Sunnerhagen

**Affiliations:** 1 Department of Community Medicine and Rehabilitation Umeå University Umeå Sweden; 2 Department of Radiation Sciences, Radiation Physics, Biomedical Engineering Umeå University Umeå Sweden; 3 Department of Epidemiology and Global Health Umeå University Umeå Sweden; 4 Department of Medical Sciences, Neurology Akademiska Sjukhuset Uppsala University Uppsala Sweden; 5 Department of Neuroscience and Physiology Sahlgrenska University Hospital Gothenburg University Gothenburg Sweden

**Keywords:** stroke, rehabilitation, needs assessment, outcome assessment, structured follow-up: follow-up, digital tool, digital health, eHealth, feasibility, randomized controlled trial, RCT, adherence, acceptability, clinical setting, Rankin scale, outpatient

## Abstract

**Background:**

Stroke is a leading cause of disability among adults, with heavy social and economic burden worldwide. A cost-effective solution is urgently needed to facilitate the identification of individual rehabilitation needs and thereby provide tailored rehabilitations to reduce disability among people who have had a stroke. A novel digital graphic follow-up tool Rehabkompassen has recently been developed to facilitate capturing the multidimensional rehabilitation needs of people who have had a stroke.

**Objective:**

The aim of this study was to evaluate the feasibility and acceptability of conducting a definitive trial to evaluate Rehabkompassen as a digital follow-up tool among people who have had a stroke in outpatient clinical settings.

**Methods:**

This pilot study of Rehabkompassen was a parallel, open-label, 2-arm prospective, proof-of-concept randomized controlled trial (RCT) with an allocation ratio of 1:1 in a single outpatient clinic. Patients who have had a stroke within the 3 previous months, aged ≥18 years, and living in the community were included. The trial compared usual outpatient visits with Rehabkompassen (intervention group) and without Rehabkompassen (control group) at the 3-month follow-up as well as usual outpatient visit with Rehabkompassen at the 12-month follow-up. Information on the recruitment rate, delivery, and uptake of Rehabkompassen; assessment and outcome measures completion rates; the frequency of withdrawals; the loss of follow-up; and satisfaction scores were obtained. The key outcomes were evaluated in both groups.

**Results:**

In total, 28 patients (14 control, 14 Rehabkompassen) participated in this study, with 100 patients screened. The overall recruitment rate was 28% (28/100). Retention in the trial was 86% (24/28) at the 12-month follow-up. All participants used the tool as planned during their follow-ups, which provided a 100% (24/24) task completion rate of using Rehabkompassen and suggested excellent feasibility. Both patient- and physician-participants reported satisfaction with the instrument (19/24, 79% and 2/2, 100%, respectively). In all, 2 (N=2, 100%) physicians and 18 (N=24, 75%) patients were willing to use the tool in the future. Furthermore, modified Rankin Scale as the primary outcome and various stroke impacts as secondary outcomes were both successfully collected and compared in this study.

**Conclusions:**

This study demonstrated the high feasibility and adherence of the study protocol as well as the high acceptability of Rehabkompassen among patients who have had a stroke and physicians in an outpatient setting in comparison to the predefined criterion. The information collected in this feasibility study combined with the amendments of the study protocol may improve the future definitive RCT. The results of this trial support the feasibility and acceptability of conducting a large definitive RCT.

**Trial Registration:**

ClinicalTrials.gov NCT04915027; https://clinicaltrials.gov/ct2/show/NCT04915027

## Introduction

Stroke is the third-leading cause of disability among adults worldwide, with heavy burden for the patients and their families as well as society [1,2]. Recently, a Global Burden of Disease report indicated that there were 143 million disability-adjusted life-years due to stroke globally in 2019 [3]. People who have had a stroke often have heterogenous functional impairments and limitations of various daily and social activities followed by decreased health-related quality of life long after stroke onset. Despite the recommendations by recent Swedish stroke guidelines, structured follow-up to identify patients’ rehabilitation needs and provide patient-tailored and precision rehabilitation regimens remains largely lacking in current stroke care [4]. Establishing such care, however, might lead to extra burden for our already time- and resource-constrained health care system. Thus, a cost-effective solution is urgently needed to facilitate identifying individual rehabilitation needs and thereby provide patient-tailored rehabilitation to reduce disability among people who have had a stroke.

To meet these challenges, we developed Rehabkompassen, a novel digital follow-up tool [5], based on well-validated, patient-reported outcome measures (PROMs). The PROMs used as Rehabkompassen questionnaires consisted of the simplified modified Rankin Scale questionnaire (smRSq), Fatigue Assessment Scale (FAS), Eating Assessment Tool (EAT-10), Hospital Anxiety and Depression Scale (HADS), Stroke Impact Scale (SIS) 3.0 plus, and 3 levels EQ-5D (EQ-5D-3L). Rehabkompassen identifies and graphically visualizes the multidimensional rehabilitation needs of patients who have had a stroke at the individual and group levels. The tool can be used as a screening tool for initial triage *before* the visit, as a communication platform *during* the visit, and as a support tool for patient referral *after* the visit. The tool allows serial assessment and may also be used as an evaluation tool *after* the eventual rehabilitation regimens have been delivered or as an illustration of the alterations of rehabilitation needs over time [5]. Both the paper and digital version of the instrument have previously been proven to be feasible, useful, and time-saving tools for the identification of unmet rehabilitation needs among persons who have had a stroke [5,6] or transient ischemic attack [7,8] in clinical practice.

Before starting a large randomized controlled trial (RCT), recruitment and retention rates, the acceptability of the intervention, and adherence to protocol need to be clarified. The aim of this study was to evaluate the feasibility and acceptability of Rehabkompassen as a digital follow-up tool in the outpatient clinic, in comparison to the control group.

## Methods

### Study Design, Setting, and Randomization

A parallel, open-label, 2-arm prospective, proof-of-concept pilot RCT with an allocation ratio of 1:1 was carried out in an outpatient clinical setting at the Department of Neurological Rehabilitation, University Hospital of Umeå, Sweden, from July 2020 to December 2021.

All participants received 2 outpatient visits at 3 and 12 months after stroke onset. At the 3-month follow-up, the participants were randomized into the intervention (an outpatient visit with Rehabkompassen) or control (an outpatient visit without Rehabkompassen) group according to a computer-generated randomization list prior to the study start. At the 12-month follow-up, all participants received an outpatient visit with Rehabkompassen.

This pilot study together with the planned definitive RCT was registered in ClinicalTrials.gov (NCT04915027). The reporting of this feasibility study was based on CONSORT (Consolidated Standards of Reporting Trials) guidelines [9].

### Ethics Approval

Ethical approvals were obtained from the regional Ethical Review Board in Umeå, Sweden (Dnr 2015/144-31) and completed (Dnr 2019-02830).

### Eligibility Criteria for Participants

All patients with a stroke diagnosis between July 2020 and March 2021 were assessed for study eligibility. Inclusion criteria were (1) aged >18 years, (2) a stroke at least 3 months prior to an outpatient visit, and (3) living in the community. Exclusion criteria were (1) inability to answer the evaluation questions; (2) inability to see the Rehabkompassen graph; and (3) lack of a BankID (a Swedish digital authorization tool), since patients without BankID prior to participating in the study were often digitally naive.

**Figure 1:**
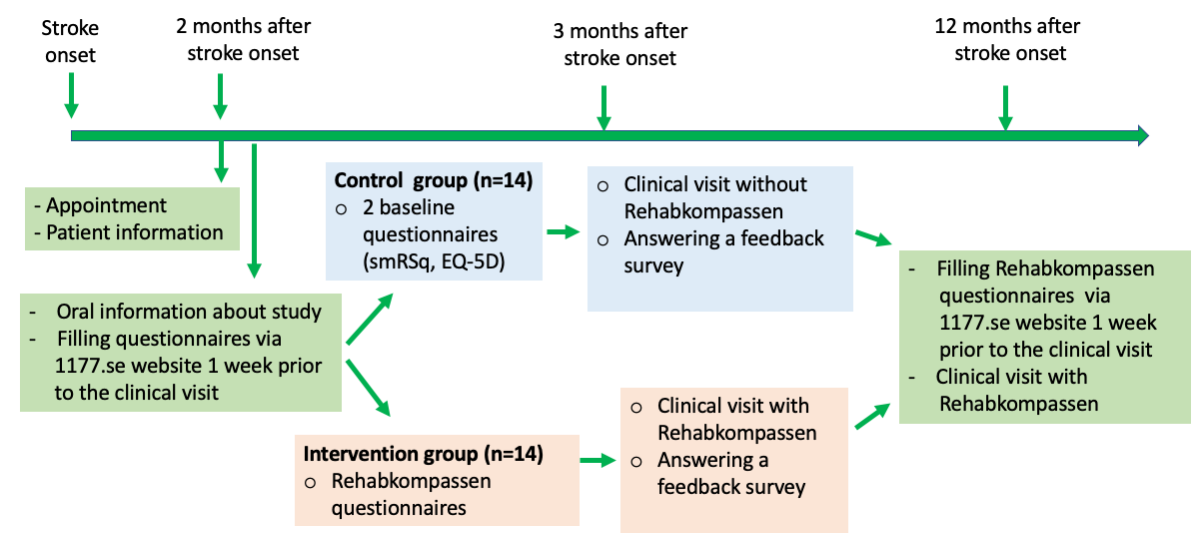
Process of the patient participants under the study. smRSq: simplified modified Rankin Scale questionnaire.

All patients who met the inclusion criteria, together with the appointment for the usual outpatient follow-up, received an invitation to the study around 2 months after stroke onset (Figure 1). The randomization list was created by an independent statistician, who was not involved in outcome assessment or the patients’ treatment. Patients who gave their consent were contacted via telephone by a research staff member at the clinic to provide oral information about the study and receive their randomized information. Written consent was obtained from all participants before participation in the study.

### 3-Month Follow-up

The intervention group consisted of Rehabkompassen and the usual outpatient follow-up that included the patient’s history of disease, examination, and rehabilitation treatment plan*.* Around 2 months after stroke onset, the patient-participants in the intervention group received the Rehabkompassen questionnaires in their inbox at the 1177.se website, which is a Swedish government-issued digital platform for citizens’ health care as described in the previous study [5]. The patient-participants filled in the Rehabkompassen questionnaires [6] at home by clicking on the links in their email inbox at the 1177.se website. The Rehabkompassen questionnaires had to be answered no later than 1 week prior to the 3-month follow-up visit (Figure 1).

**Figure 2:**
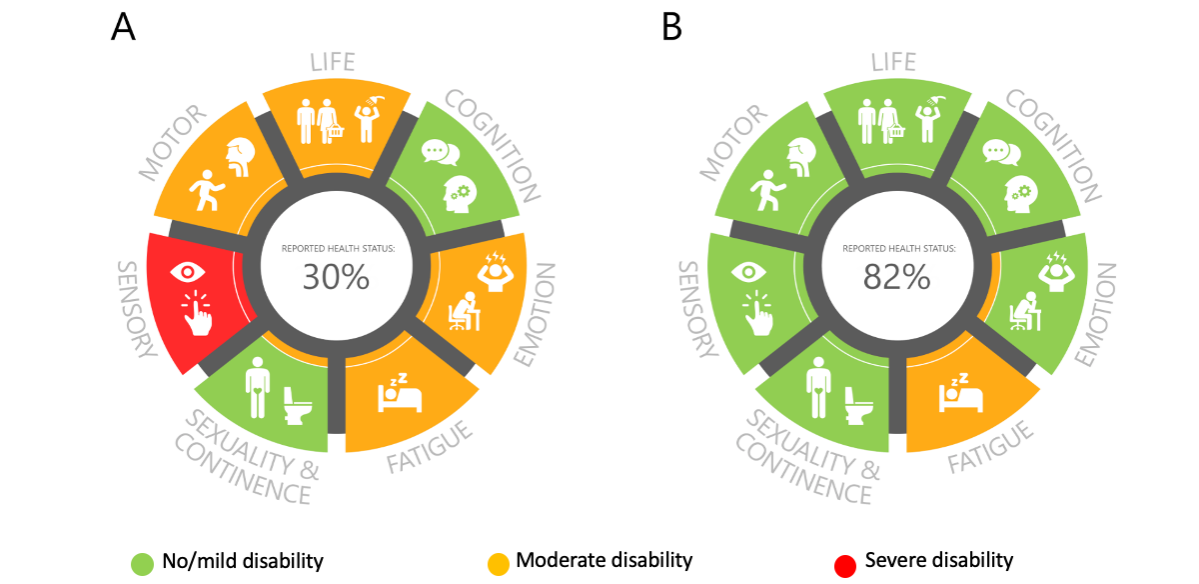
Examples of the Rehabkompassen graphs of a patient who has had a stroke showing (A) more rehabilitation needs at the 3-month follow-up and (B) fewer rehabilitation needs at the 12-month follow-up. The Rehabkompassen graphs (A and B) show the self-reported health status of the patient who has had a stroke in a color-coded holistic view with 7 commonly affected areas by stroke: life, cognition, emotion, fatigue, sexuality and continence, sensory function, and motor function. Each area consists of several domains. An extra color-coded field in the inner edge of each area represents the lowest function value in the area.

Prior to the 3-month follow-up, a nurse prioritized the team recourse based on the patient’s Rehabkompassen data. During the follow-up visit, a doctor showed the patient’s personal Rehabkompassen graph (see Figure 2A for an example at the 3-month follow-up) on the computer and used it as an illustration to discuss with patients their health status and rehabilitation needs.

The control group received the usual follow-up without Rehabkompassen with otherwise identical procedures as the intervention group. To collect the baseline data, the control group filled in only 2 questionnaires (smRSq and EQ-5D-3L) via the 1177.se website prior to their follow-up appointments (Figure 1).

The length of each visit in both intervention and control groups was the same—approximately 45 minutes. After the visit, all participants in both intervention and control groups received various rehabilitation regimens based on their rehabilitation needs.

### 12-Month Follow-up

All participants from the control and intervention groups filled in the Rehabkompassen questionnaires via the 1177.se website at home 1 week prior to a 12-month follow-up visit (Figure 1). The patients’ Rehabkompassen graphs were used in combination with the usual outpatient follow-up as described above for the intervention group. The Rehabkompassen graph at the 3-month follow-up (see Figure 2A for an example for those in the intervention group) could be used as an evaluation tool to compare with the Rehabkompassen graph at the 12-month follow-up (Figure 2B).

### Postvisit Assessments of Satisfaction With the Rehabkompassen Tool

Acceptability was assessed in terms of the patient-participants’ satisfaction with Rehabkompassen. After the 3- and 12-month follow-up visits, all patient-participants in both intervention and control groups answered a satisfaction questionnaire through the 1177.se website. The questionnaire addressed their overall experiences of the conversation with the physician and their satisfaction of using the Rehabkompassen graph during the follow-up visit. The satisfaction of Rehabkompassen was rated in terms of how it affected their ability to understand their rehabilitation needs during the consultation throughout the outpatient visit by using a Likert scale from 1 (very easy) to 5 (very difficult). Participants rating either very easy or fairly easy were considered as satisfied with the tool. The patients’ satisfaction rate was calculated by the number of patients who were satisfied with the tool divided by the total number of patient-participants.

At the end of the 3-month follow-ups, 2 physicians involved in the study answered a questionnaire with 5 questions regarding the different aspects of utility to provide feedback on the perceived feasibility and satisfaction of the instrument in clinical practice. A Likert scale ranging from 1 (strongly disagree) to 5 (strongly agree) was used, with higher scores indicating better outcomes. Ratings of strongly agree and fairly agree were considered as the physicians being satisfied with the tool. The physicians’ satisfaction rate was calculated by the total number of satisfied aspects divided by the total number of aspects.

After analyzing the 3- and 12-month postvisit assessments, we realized that several patient-participants did not fully understand what the Rehabkompassen graph was despite being satisfied with their outpatient visits with Rehabkompassen. Therefore, we amended the questions regarding the graph and added a simple picture of a Rehabkompassen graph to help the participants recall and more easily understand the question. This revised questionnaire will be used in the future definitive RCT.

### Outcomes

#### Feasibility Information

To study the feasibility of the study, information on the recruitment rate, adherence, delivery, and uptake of the Rehabkompassen; satisfaction; and possible future use were collected in this study [10,11]. We predefined the following thresholds for specific feasibility and acceptability criteria for deciding whether to progress to the next stage (ie, to carry out the future definitive RCT): (1) the patient recruitment rate would be >20% of the total number of patients who were asked to participate in the study; (2) adherence to the study protocol would be >60% of the total number of the participants with a written consent; (3) the feasibility (delivery and use) of Rehabkompassen would be >60% of the total number of patients using Rehabkompassen as planned; (4) the acceptability of Rehabkompassen (the mean level of satisfaction from both patients and physicians) would be >60% of the total participants; and (5) willingness to use the tool in the future would be >60% of the total participants. However, not reaching the predefined criteria does not necessarily indicate the unfeasibility of the trial but rather underlines that some changes to the protocol would be needed.

#### Primary Outcome

The smRSq [12-14] was used to collect the primary outcome of the modified Rankin Scale (mRS) that measures patients’ independence or disability level in their daily activities. The smRSq is based on the yes-or-no responses to 5 questions, which is then used to calculate the mRS score ranging from 0 to 5 [12]. A favorable outcome was defined as an mRS score of 0-2 (from no symptoms to independent but with minor disability). A poor outcome was defined as an mRS score of 3-5 (from disability but able to walk to bed-bound and in need of full nursing care) or 6 (death). The completion rates, variances, and 95% CIs for the difference between the intervention arms were analyzed.

#### Secondary Outcomes

Secondary outcomes were assessed and collected directly after the patients filled in the Rehabkompassen questionnaires.

Fatigue was measured by FAS [15], a questionnaire used for identifying symptoms of chronic fatigue. It is comprised of 10 questions regarding both physical and mental fatigue answered on a scale from 1 (never) to 5 (always).

Dysphagia was assessed by EAT-10 [16] including 10 questions concerning swallowing difficulties. Each question is to be answered on a scale from 0 (no problem) to 4 (severe problem).

Depression and anxiety were measured by HADS [17], a screening tool for the assessment of anxiety and depression. It comprises 7 questions about anxiety and 7 questions about depression, answered on a scale from 0 (no symptoms) to 3 (severe symptoms). The subscales for anxiety and depression were added and interpreted separately.

Stroke impacts were assessed by SIS [18], a patient-reported, stroke-specific outcome measurement containing 59 questions and a visual analog scale for the estimation of perceived stroke recovery. As secondary outcomes, this study assessed stroke impacts within 9 domains, namely strength, memory/cognition, feelings/emotions, communication, personal activities of daily living, instrumental activities of daily living, mobility, motor impact, and social participation. In the previous study, we also added items covering continence and sexual function as well as sleep disturbance, which was named SIS-plus [6]. The SIS data are presented in ordinal scores ranging from 0 to 100, with higher scores indicating less impact of stroke [18].

Health-related quality of life and cost-effectiveness were measured by EQ-5D-3L [19,20]. The EQ-5D-3L consists of 2 parts: a visual analog scale and a descriptive system covering 5 dimensions of health (mobility, hygiene, usual activities, pain/discomfort, and anxiety/depression) with 3 response alternatives (ranging from no problems to extreme problems). The latter can be translated to an index value with anchor points 0 (death) and 1 (full health) for eliciting the overall health utility score, corresponding to a quality-adjusted life-years score.

### Data Presentation and Statistics

Descriptive statistics were presented with mean and median values, SDs, quartiles, and proportions. The recruitment rate was calculated by the number of the participants in each group divided by the total number of patients who were assessed for eligibility. The other remaining rate was calculated by the number of the participants in each criterion divided by the number of the patient recruited in its group. In the Rehabkompassen graph, PROMs scale data were converted to a scale from 0 (worst outcome) to 100 (best outcome) but unchanged in terms of variable properties [6].

Although no statistically significant difference is expected to be found in this feasibility study, the differences on the primary and secondary outcomes on an ordinal scale at the 12-month follow-up between the intervention and control groups were tested using ordinal logistic regression.

All data were analyzed using SPSS software (version 26.0; IBM Corp). The figures were generated by GraphPad Prism software (version 9; Dotmatics). A 2-tailed *P* value of <.05 was considered significant.

## Results

### Patient Recruitment and Feasibility Assessments

**Figure 3:**
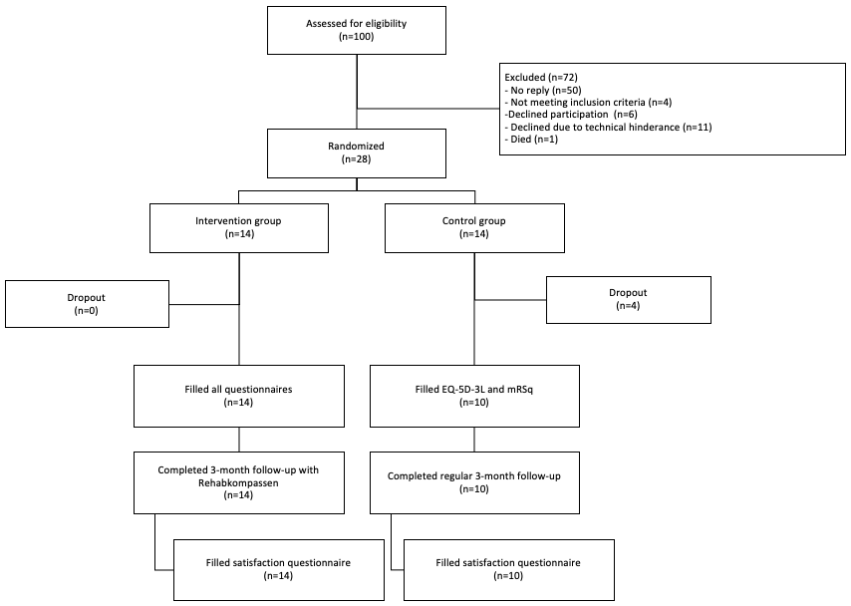
Flowchart of participant recruitment, randomization, and retention. EQ-5D-3L: 3 levels EQ-5D; smRSq: simplified modified Rankin Scale questionnaire.

In all, 100 patients were assessed for eligibility (Figure 3) from July 2020 to March 2021, to a high extent coinciding with the second wave of COVID-19 in Sweden. Of these 100 patients, 28 participants gave written consent, which equated to a recruitment rate of 28%. Among the 72 patients who did not participate in the study (Figure 3), 50 patients never responded to the study invitation letter; 4 patients did not meet inclusion criteria; 6 patients declined without giving a reason; and 1 patient died. The remaining 11 (N=100, 11%) patients declined participation due to various technical issues, such as no computer at home, no internet, no BankID, or inability to use these technologies.

Of the 14 participants in the control group, 4 dropped out at 3-month follow-up, with no dropout in the intervention group (Figure 3), resulting in a total trial completion rate of 86% (24/28), which was higher than the predefined adherence cutoff (>60%) in the study protocol (Table 1).

All 14 participants in the intervention group at the 3-month follow-up and all 24 participants at the 12-month follow-up used Rehabkompassen, which resulted in 100% (24/24) on the feasibility of the instrument. This was much better than the predefined feasibility cutoff (>60%; Table 1). Satisfaction with the tool was reported among 79% (19/24) of the patients and 100% (2/2) of the physicians. Moreover, 75% (18/24) of patients and both (2/2, 100%) physicians would prefer to use the tool in the future (Table 1).

**Table 1:** Criteria for the feasibility of Rehabkompassen.

Criterion (predefined cutoff, %)		Intervention group, n (%)	Control group, n (%)	Total, n (%)
Recruitment (total >20%; N=100)		14 (14)	14 (14)	28 (28)
**Adherence (total >60%; intervention group: n=14; control group: n=14; total: n=28)**				
	Dropout at the 3-month follow-up	0 (0)	4 (29)	4 (14)
	Dropout at the 12-month follow-up	0 (0)	0 (0)	0 (0)
	Adherence of study protocol or retention in the study	14 (100)	10 (71)	24 (86)
**Feasibility (total >60%; intervention group: n=14; control group: n=14; total: n=28)**				
	Delivery of Rehabkompassen at the 3-month follow-up	14 (100)	N/A^a^	14 (100)
	Delivery of Rehabkompassen at the 12-month follow-up	14 (100)	10 (100)	24 (100)
**Acceptability or uptake (total >60%)**				
	Patients’ satisfaction of Rehabkompassen (n=24)	N/A	N/A	19 (79)
	Physicians’ satisfaction of Rehabkompassen (n=2)	N/A	N/A	2 (100)
**Use in the future (total >60%)**				
	Patients’ willingness to use Rehabkompassen (n=24)	N/A	N/A	18 (75)
	Physicians’ willingness to use Rehabkompassen (n=2)	N/A	N/A	2 (100)

^a^N/A: not applicable.

### Participant Characteristics

The participants’ mean age was 68 years in the intervention group and 66 years in the control group (Table 2). Of the 24 participants, the majority (n=13, 54%) were male. All (n=24, 100%) participants had at least completed primary school, with 50% (n=12) having university degrees, possibly due to the catchment area being a university city. There were 2 (8%) participants who identified their computer skills as beginner level, whereas the other participants rated their computer skills as average or good. A majority (n=22, 92%) had previous experience with the 1177.se website, whereas 2 (8%) had never logged onto the platform. There were no significant differences in characteristics between the intervention and control groups.

**Table 2:** Baseline characteristic of the patient-participants.

Characteristic, category			Intervention group, (n=14)		Control group, (n=10)
Age (year), mean (SD)			68 (12.0)		66 (11.7)
Sex, female, n (%)			5 (36)		6 (60)
Modified Rankin Scale score, median (IQR)			0 (0-1.25)		1 (0-1)
**Highest education, n (%)**					
	No completed education	0 (0)		0 (0)	
	Primary school or equivalent	3 (21)		2 (20)	
	High school or equivalent	3 (21)		4 (40)	
	University or college	8 (57)		4 (40)	
**Perceived computer skills, n (%)**					
	Beginner	2 (14)		0 (0)	
	Average	6 (43)		6 (60)	
	Good	6 (43)		4 (40)	
	Expert	0 (0)		0 (0)	

### Assessment of Satisfaction Among Physicians

**Figure 4:**
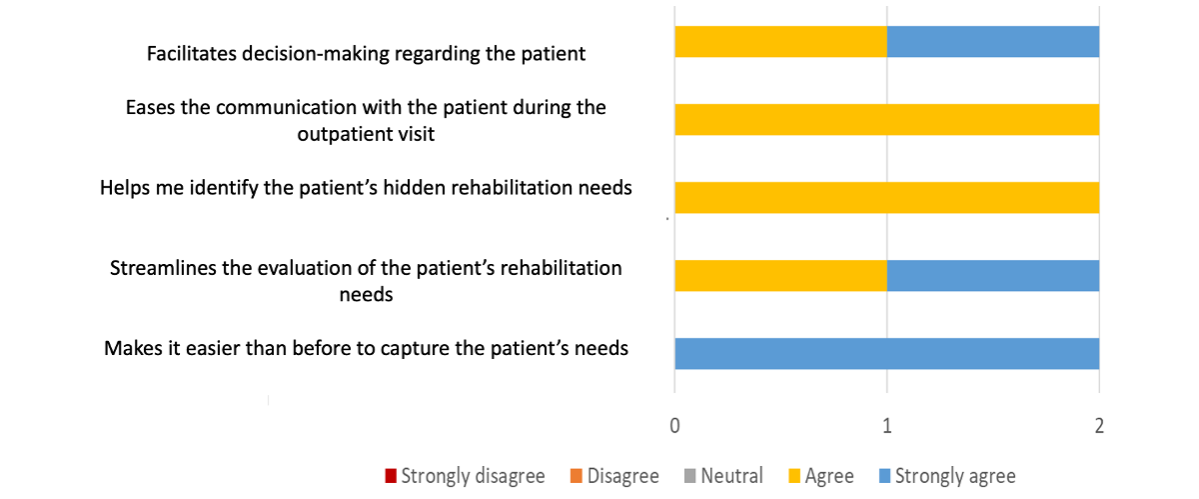
The 2 physicians’ positive feedback on using the Rehabkompassen tool.

In total, 2 physicians (one of them is a senior consultant physiatrist) participated in the pilot study to explore the feasibility and satisfaction questions. Both physicians were very positive regarding the potential usefulness of the Rehabkompassen tool (Figure 4). They reported that the tool facilitated the identification of rehabilitation needs and streamlined evaluation and decision-making regarding the patients’ rehabilitation needs. They agreed that the tool made it easier to communicate with their patients and avoid overlooking hidden symptoms. Both (2/2, 100%) physicians would like to use the instrument in the future, which indicated a higher acceptability than the predefined cutoff at 60%.

### Primary Outcome

**Figure 5:**
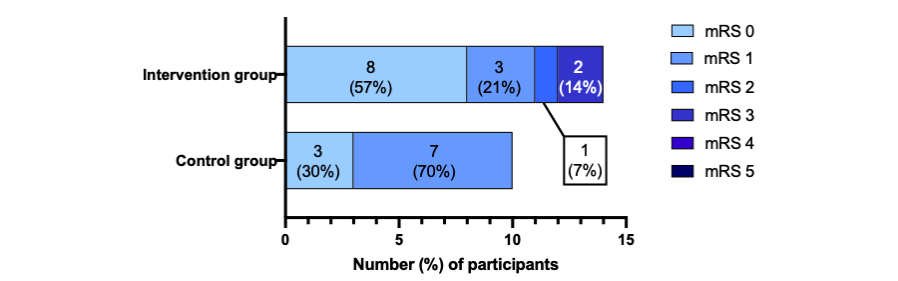
Distribution of mRS scores demonstrating no significant difference between the intervention and control groups. mRS: modified Rankin Scale.

The mRS score at the 12-month follow-up was analyzed in 24 participants, of which 14 patients were allocated to the intervention group with Rehabkompassen and 10 patients were allocated to the control group without Rehabkompassen at the 3-month follow-up. An ordinal comparison of the distribution of patients across mRS categories at 12 months demonstrated no statistically significant difference between the groups (odds ratio 0.429, 95% CI –1.979 to 1.120; *P*=.59; Figure 5).

### Secondary Outcomes

A panoramic view of various stroke impacts among patients in the intervention and control groups are presented in Table 3. Briefly, the most severe problems reported in median (IQR) by the intervention group were fatigue at 69 (32-89), strength at 72 (50-96), sexual dysfunction at 75 (44-100), and participation at 75 (55-100). In the control group, the most reported problems were strength at 62 (50-100), quality of life at 72 (65-100), pain at 75 (50-100), and daily activity at 80 (80-100). There were no significant differences on each stroke impact between the intervention and control groups (all *P*>.05; see Table 3).

**Table 3:** Extent of rehabilitation needs identified by Rehabkompassen at the 12-month follow-up in both intervention and control groups. The different conditions were assessed by various instruments and grouped into different domains. The extent of rehabilitation needs scores range from 0 (worst outcome or unmet rehabilitation need) to 100 (best outcome or no rehabilitation needs).

Domain, condition (instrument)			Intervention group, median (IQR)		Control group, median (IQR)		*P* value
**Social participation**							
	Activities of daily living or instrumental activities of daily living (SIS^a^)	94 (82-100)		98 (94-100)		.24	
	Activity (mRS^b^)	100 (75-100)		80 (80-100)		.59	
	Participation (SIS)	75 (55-100)		96 (83-100)		.18	
	Quality of life (EQ-5D)	79 (20-100)		72 (65-100)		.76	
	Visual analog scale (EQ-5D)	82 (48-92)		75 (61-92)		>.99	
**Cognition**							
	Communications (SIS)	96 (86-100)		96 (95-100)		.32	
	Memory and thinking (SIS)	91 (80-100)		98 (93-100)		.16	
**Emotion**							
	Depression (HADS^c^)	91 (70-100)		94 (89-97)		.65	
	Anxiety (HADS)	85 (70-100)		87 (81-97)		.78	
	Anxiety (GAD^d^)	90 (75-100)		90 (83-100)		.61	
**Fatigue**							
	Fatigue (FAS^e^)	69 (32-89)		81 (62-93)		.25	
	Sleep (SIS+)	79 (58-94)		88 (75-100)		.39	
**Continence and sexual function**							
	Bladder (SIS+)	100 (88-100)		92 (83-100)		.55	
	Bowel (SIS+)	100 (73-100)		100 (88-100)		.57	
	Sexual dysfunction (SIS+)	75 (44-100)		88 (56-100)		.66	
**Sensory function**							
	Vision (SIS+)	93 (80-100)		93 (79-100)		.80	
	Smell (SIS)	100 (50-100)		100 (69-100)		.90	
	Taste (SIS+)	100 (50-100)		100 (69-100)		.56	
	Hearing (SIS+)	100 (50-100)		100 (94-100)		.29	
	Sensory (SIS+)	100 (75-100)		100 (88-100)		.95	
	Pain (SIS+)	88 (25-100)		75 (50-100)		.75	
**Motor function**							
	Stiffness (SIS+)	88 (25-100)		88 (50-100)		.99	
	Strength (SIS)	72 (50-96)		62 (50-100)		.74	
	Mobility (SIS)	93 (77-100)		100 (89-100)		.14	
	Hand function (SIS)	98 (64-100)		100 (89-100)		.31	
	Swallow function (EAT-10^f^)	100 (66-100)		100 (98-100)		.38	

^a^SIS: Stroke Impact Scale.

^b^mRS: modified Rankin Scale.

^c^HADS: Hospital Anxiety and Depression Scale.

^d^GAD: Generalized Anxiety Disorder.

^e^FAS: Fatigue Assessment Scale.

^f^EAT-10: Eating Assessment Tool.

## Discussion

### Principal Findings

This randomized clinical feasibility study investigated the feasibility and acceptability of conducting a definitive trial evaluating Rehabkompassen as a digital follow-up tool among persons who have had a stroke in an outpatient clinic setting. The overall recruitment rate was 28%. Retention in the trial was 86% at the 12-month follow-up, which indicated high adherence to the study protocol. Additionally, a 100% task completion rate of using Rehabkompassen in the study suggested excellent feasibility of the tool. Satisfactions with the instrument reported by both patient- and physician-participants (79% and 100%, respectively) showed the high acceptability of Rehabkompassen and the willingness to use the tool in the future. Furthermore, both mRS as the primary outcome and various stroke impacts as secondary outcomes were successfully collected and compared in this study.

The feasibility of conducting a definitive trial evaluating Rehabkompassen in this study was assessed on recruitment rate, retention rate, and the delivery and uptake of the Rehabkompassen tool. The recruitment rate of 28% was slightly above our predefined cutoff. However, we hope for a better recruitment rate in the future definitive RCT, since this feasibility study was carried out during a heavy COVID-19 pandemic period in Sweden. Compared to the predefined cutoff at 60%, the retention rate of 86% in this study implies that the study protocol was well tolerated by both patients and clinicians. High-quality data may be collected if we achieve a similar retention rate with fewer missing values in the future definitive RCT. Together with a 100% task completion rate of using Rehabkompassen, these excellent feasibility data support our plan of conducting a large definitive RCT.

The acceptability of the tool by both patients (79%) and physicians (100%) could partly explain the higher retention rate in the study compared to the predefined cutoff at 60%. Furthermore, the doctors reported that the tool facilitated communication with their patients and helped identify hidden symptoms, which is partly congruent with feedback from the patients. The satisfaction among the end users is consistent with our previous findings where the usability of the instrument is well demonstrated [5], which is also supported by the high willingness to use the Rehabkompassen tool in the future.

The differences of mRS observed between the treatment arms in this pilot study were not statistically significant, which is consistent with other large RCTs where mRS was chosen as the primary outcome [13,21]. Although no statistically significant difference on mRS as the primary outcome is expected to be found in this feasibility study, the results still raised a critical concern of whether the mRS as a single primary outcome was sensitive enough to capture the subtle alterations of treatment-effects in the future definitive RCT. Additionally, the background characteristics of the participants demonstrated that most of the target study population had a mild to moderate disability with more limitation on social participation, which is in line with previous Swedish stroke RCTs [13,22]. To catch the minor but important changes on both daily activity and social participation over time, we added Domain 8 in SIS [23] as another primary outcome to use in the future definitive RCT, since mRS covers mainly daily activity [14].

This randomized feasibility study revealed other concerns in addition to the abovementioned amendments, such as the extension of exclusion criteria with BankID and revision on the satisfaction questionnaire. We found that 11% of patients without sufficient computer knowledge were excluded in the study, which provided information on how many patients would need extra help in case such persons would like to participate in the future definitive study. Even with multiple secondary outcomes collected in this study, it was considered less time-consuming for the health care professionals, since these outcomes were based upon PROMs filled out in advance by the patients through Rehabkompassen. Thus, this would not jeopardize the collection of primary outcome data.

Although this feasibility study provides important information and necessary amendments for the future definitive RCT study, there were a couple of limitations. Since this feasibility study was carried out in only 1 outpatient clinic, we cannot generalize the results directly to different participating clinics with various clinical routines in the future multicenter RCT. It remains a challenge to fit the Rehabkompassen tool within various existing clinical routines despite the high rates of feasibility and acceptability demonstrated in this study. Furthermore, this feasibility study was performed by an experienced clinical research team, which is crucial for reaching a high-quality study. Therefore, it is very important that knowledge transfer, timely troubleshooting, and problem-solving by the experienced research team be available during the future definitive RCT. At this stage, the results remain difficult to generalize due to its limited sample size (2 physicians; and 14 patients in the control and intervention groups, respectively) in this pilot study; thus, a further definitive RCT is needed.

### Conclusions

This study demonstrated very high feasibility and adherence to the study protocol as well as the high acceptability of the Rehabkompassen tool among people who have had a stroke and physicians in an outpatient setting in comparison to the predefined criterion. This information may improve the future definitive RCT. The results of this trial support the feasibility and acceptability of conducting a large definitive RCT.

## Appendix

Multimedia Appendix 1 CONSORT-eHEALTH checklist (V 1.6.1).

## Abbreviations

CONSORT: Consolidated Standards of Reporting Trials
EAT-10: Eating Assessment Tool
EQ-5D-3L: 3 levels EQ-5D
FAS: Fatigue Assessment Scale
HADS: Hospital Anxiety and Depression Scale
mRS: modified Rankin Scale
PROM: patient-reported outcome measure
RCT: randomized controlled trial
SIS: Stroke Impact Scale
smRSq: simplified modified Rankin Scale questionnaire

## Data Availability

The data sets generated and/or analyzed during the current study are available from the corresponding author on reasonable request.
