# Supplementary material for: Digital Graphic Follow-up Tool (Rehabkompassen) for Identifying Rehabilitation Needs Among People After Stroke: Randomized Clinical Feasibility Study
Source: JMIR Hum Factors. 2022 Jul 29;9(3):e38704. doi: 10.2196/38704 (PMC9377427; doi:10.2196/38704)
Supplement: Multimedia Appendix 1 [file humanfactors_v9i3e38704_app1.pdf]

CONSORT-EHEALTH (V 1.6.1) - Submission/Publication Form

The CONSORT-EHEALTH checklist is intended for authors of randomized trials evaluating web-based and Internet-based applications/interventions, including mobile interventions, electronic games (incl multiplayer games), social media, certain telehealth applications, and other interactive and/or networked electronic applications. Some of the items (e.g. all subitems under item 5 - description of the intervention) may also be applicable for other study designs.

The goal of the CONSORT EHEALTH checklist and guideline is to be
a) a guide for reporting for authors of RCTs,
b) to form a basis for appraisal of an ehealth trial (in terms of validity)

CONSORT-EHEALTH items/subitems are MANDATORY reporting items for studies published in the Journal of Medical Internet Research and other journals / scientific societies endorsing the checklist.

Items numbered 1., 2., 3., 4a., 4b etc are original CONSORT or CONSORT-NPT (non-pharmacologic treatment) items.
Items with Roman numerals (i., ii, iii, iv etc.) are CONSORT-EHEALTH extensions/clarifications.

As the CONSORT-EHEALTH checklist is still considered in a formative stage, we would ask that you also RATE ON A SCALE OF 1-5 how important/useful you feel each item is FOR THE PURPOSE OF THE CHECKLIST and reporting guideline (optional).

Mandatory reporting items are marked with a red *.
In the textboxes, either copy & paste the relevant sections from your manuscript into this form - please include any quotes from your manuscript in QUOTATION MARKS,
or answer directly by providing additional information not in the manuscript, or elaborating on why the item was not relevant for this study.

YOUR ANSWERS WILL BE PUBLISHED AS A SUPPLEMENTARY FILE TO YOUR PUBLICATION IN JMIR AND ARE CONSIDERED PART OF YOUR PUBLICATION (IF ACCEPTED).
Please fill in these questions diligently. Information will not be copyedited, so please use proper spelling and grammar, use correct capitalization, and avoid abbreviations.

DO NOT FORGET TO SAVE AS PDF _AND_ CLICK THE SUBMIT BUTTON SO YOUR ANSWERS ARE IN OUR DATABASE !!!

Citation Suggestion (if you append the pdf as Appendix we suggest to cite this paper in the caption):
Eysenbach G, CONSORT-EHEALTH Group
CONSORT-EHEALTH: Improving and Standardizing Evaluation Reports of Web-based and Mobile Health Interventions
J Med Internet Res 2011;13(4):e126
URL: http://www.jmir.org/2011/4/e126/
doi: 10.2196/jmir.1923
PMID: 22209829

xihu6902@gmail.com (Delas inte) Byt konto

Utkastet har inte sparats

*Obligatorisk

Your name *

First Last

Xiaolei Hu

Primary Affiliation (short), City, Country *

University of Toronto, Toronto, Canada

Umeå University, Umeå, Sweden

Your e-mail address *

abc@gmail.com

xiaolei.hu@umu.se

Title of your manuscript *

Provide the (draft) title of your manuscript.

A Digital Graphic Follow-up Tool (Rehabkompassen®) for Identifying Rehabilitation Needs Among People With Stroke: Randomized Clinical Feasibility Study

Name of your App/Software/Intervention *

If there is a short and a long/alternate name, write the short name first and add the long name in brackets.

Rehabkompassen®

Evaluated Version (if any)

e.g. "V1", "Release 2017-03-01", "Version 2.0.27913"

Version 1.0

Language(s) *

What language is the intervention/app in? If multiple languages are available, separate by comma (e.g. "English, French")

Swedish, English

URL of your Intervention Website or App

e.g. a direct link to the mobile app on app in appstore (itunes, Google Play), or URL of the website. If the intervention is a DVD or hardware, you can also link to an Amazon page.

We have no URL for the Rehabkompassen

Måste vara en giltig webbadress

URL of an image/screenshot (optional)

We have no URL for the Rehabkompassen

Måste vara en giltig webbadress

Accessibility *

Can an enduser access the intervention presently?

- ○ access is free and open
- ◉ access only for special usergroups, not open
- ○ access is open to everyone, but requires payment/subscription/in-app purchases
- ○ app/intervention no longer accessible
- ○ Övrigt:

Primary Medical Indication/Disease/Condition *

e.g. "Stress", "Diabetes", or define the target group in brackets after the condition, e.g. "Autism (Parents of children with)", "Alzheimers (Informal Caregivers of)"

Stroke

Primary Outcomes measured in trial *

comma-separated list of primary outcomes reported in the trial

To evaluate feasibility and acceptability of con

Secondary/other outcomes

Are there any other outcomes the intervention is expected to affect?

the key outcomes in the large definitive RCT will be assessed in both intervention and control groups in the current pilot study

Recommended "Dose" *

What do the instructions for users say on how often the app should be used?

- ○ Approximately Daily
- ○ Approximately Weekly
- ○ Approximately Monthly
- ○ Approximately Yearly
- ◉ "as needed"
- ○ Övrigt:

Approx. Percentage of Users (starters) still using the app as recommended after 3 months *

- ○ unknown / not evaluated
- ○ 0-10%
- ○ 11-20%
- ○ 21-30%
- ○ 31-40%
- ○ 41-50%
- ○ 51-60%
- ○ 61-70%
- ○ 71%-80%
- ○ 81-90%
- ◉ 91-100%
- ○ Övrigt:

Overall, was the app/intervention effective? *

- ◯ yes: all primary outcomes were significantly better in intervention group vs control
- ◯ partly: SOME primary outcomes were significantly better in intervention group vs control
- ◯ no statistically significant difference between control and intervention
- ◯ potentially harmful: control was significantly better than intervention in one or more outcomes
- ◉ inconclusive: more research is needed
- ◯ Övrigt:

Article Preparation Status/Stage *

At which stage in your article preparation are you currently (at the time you fill in this form)

- ◯ not submitted yet - in early draft status
- ◯ not submitted yet - in late draft status, just before submission
- ◯ submitted to a journal but not reviewed yet
- ◯ submitted to a journal and after receiving initial reviewer comments
- ◉ submitted to a journal and accepted, but not published yet
- ◯ published
- ◯ Övrigt:

Journal *

If you already know where you will submit this paper (or if it is already submitted), please provide the journal name (if it is not JMIR, provide the journal name under "other")

- ○ not submitted yet / unclear where I will submit this
- ○ Journal of Medical Internet Research (JMIR)
- ○ JMIR mHealth and UHealth
- ○ JMIR Serious Games
- ○ JMIR Mental Health
- ○ JMIR Public Health
- ○ JMIR Formative Research
- ◉ Other JMIR sister journal
- ○ Övrigt:

Is this a full powered effectiveness trial or a pilot/feasibility trial? *

- ◉ Pilot/feasibility
- ○ Fully powered

Manuscript tracking number *

If this is a JMIR submission, please provide the manuscript tracking number under "other" (The ms tracking number can be found in the submission acknowledgement email, or when you login as author in JMIR. If the paper is already published in JMIR, then the ms tracking number is the four-digit number at the end of the DOI, to be found at the bottom of each published article in JMIR)

- ○ no ms number (yet) / not (yet) submitted to / published in JMIR
- ◉ Övrigt: 38704

TITLE AND ABSTRACT

1a) TITLE: Identification as a randomized trial in the title

1a) Does your paper address CONSORT item 1a? *

I.e does the title contain the phrase "Randomized Controlled Trial"? (if not, explain the reason under "other")

- ◉ yes
- ○ Övrigt:

1a-i) Identify the mode of delivery in the title

Identify the mode of delivery. Preferably use “web-based” and/or “mobile” and/or “electronic game” in the title. Avoid ambiguous terms like “online”, “virtual”, “interactive”. Use “Internet-based” only if Intervention includes non-web-based Internet components (e.g. email), use “computer-based” or “electronic” only if offline products are used. Use “virtual” only in the context of “virtual reality” (3-D worlds). Use “online” only in the context of “online support groups”. Complement or substitute product names with broader terms for the class of products (such as “mobile” or “smart phone” instead of “iphone”), especially if the application runs on different platforms.

	1	2	3	4	5	
subitem not at all important	○	○	○	○	◉	essential

Rensa markering

Does your paper address subitem 1a-i? *

Copy and paste relevant sections from manuscript title (include quotes in quotation marks "like this" to indicate direct quotes from your manuscript), or elaborate on this item by providing additional information not in the ms, or briefly explain why the item is not applicable/relevant for your study

"Randomized Clinical Feasibility Study", "Digital"

1a-ii) Non-web-based components or important co-interventions in title

Mention non-web-based components or important co-interventions in title, if any (e.g., “with telephone support”).

	1	2	3	4	5	
subitem not at all important	○	○	◉	○	○	essential

Rensa markering

Does your paper address subitem 1a-ii?

Copy and paste relevant sections from manuscript title (include quotes in quotation marks "like this" to indicate direct quotes from your manuscript), or elaborate on this item by providing additional information not in the ms, or briefly explain why the item is not applicable/relevant for your study

"A Digital Graphic Follow-up Tool "

1a-iii) Primary condition or target group in the title

Mention primary condition or target group in the title, if any (e.g., “for children with Type I Diabetes”) Example: A Web-based and Mobile Intervention with Telephone Support for Children with Type I Diabetes: Randomized Controlled Trial

	1	2	3	4	5	
subitem not at all important	○	○	○	○	◉	essential

Rensa markering

Does your paper address subitem 1a-iii? *

Copy and paste relevant sections from manuscript title (include quotes in quotation marks "like this" to indicate direct quotes from your manuscript), or elaborate on this item by providing additional information not in the ms, or briefly explain why the item is not applicable/relevant for your study

"Identifying Rehabilitation Needs Among People With Stroke:"

1b) ABSTRACT: Structured summary of trial design, methods, results, and conclusions

NPT extension: Description of experimental treatment, comparator, care providers, centers, and blinding status.

1b-i) Key features/functionalities/components of the intervention and comparator in the METHODS section of the ABSTRACT

Mention key features/functionalities/components of the intervention and comparator in the abstract. If possible, also mention theories and principles used for designing the site. Keep in mind the needs of systematic reviewers and indexers by including important synonyms. (Note: Only report in the abstract what the main paper is reporting. If this information is missing from the main body of text, consider adding it)

	1	2	3	4	5	
subitem not at all important	○	○	○	○	◉	essential

Rensa markering

Does your paper address subitem 1b-i? *

Copy and paste relevant sections from the manuscript abstract (include quotes in quotation marks "like this" to indicate direct quotes from your manuscript), or elaborate on this item by providing additional information not in the ms, or briefly explain why the item is not applicable/relevant for your study

"The trial compared usual outpatient visits with Rehabkompassen® (intervention group) and without (control group) at 3-month follow-up as well as usual outpatient visit with Rehabkompassen® at 12-month follow-up"

1b-ii) Level of human involvement in the METHODS section of the ABSTRACT

Clarify the level of human involvement in the abstract, e.g., use phrases like “fully automated” vs. “therapist/nurse/care provider/physician-assisted” (mention number and expertise of providers involved, if any). (Note: Only report in the abstract what the main paper is reporting. If this information is missing from the main body of text, consider adding it)

	1	2	3	4	5	
subitem not at all important	○	○	○	○	◉	essential

Rensa markering

Does your paper address subitem 1b-ii?

Copy and paste relevant sections from the manuscript abstract (include quotes in quotation marks "like this" to indicate direct quotes from your manuscript), or elaborate on this item by providing additional information not in the ms, or briefly explain why the item is not applicable/relevant for your study

"The trial compared usual outpatient visits with Rehabkompassen® (intervention group) and without (control group) at 3-month follow-up as well as usual outpatient visit with Rehabkompassen® at 12-month follow-up"

1b-iii) Open vs. closed, web-based (self-assessment) vs. face-to-face assessments in the METHODS section of the ABSTRACT

Mention how participants were recruited (online vs. offline), e.g., from an open access website or from a clinic or a closed online user group (closed usergroup trial), and clarify if this was a purely web-based trial, or there were face-to-face components (as part of the intervention or for assessment). Clearly say if outcomes were self-assessed through questionnaires (as common in web-based trials). Note: In traditional offline trials, an open trial (open-label trial) is a type of clinical trial in which both the researchers and participants know which treatment is being administered. To avoid confusion, use “blinded” or “unblinded” to indicated the level of blinding instead of “open”, as “open” in web-based trials usually refers to “open access” (i.e. participants can self-enrol). (Note: Only report in the abstract what the main paper is reporting. If this information is missing from the main body of text, consider adding it)

	1	2	3	4	5	
subitem not at all important	○	○	○	○	◉	essential

Rensa markering

Does your paper address subitem 1b-iii?

Copy and paste relevant sections from the manuscript abstract (include quotes in quotation marks "like this" to indicate direct quotes from your manuscript), or elaborate on this item by providing additional information not in the ms, or briefly explain why the item is not applicable/relevant for your study

"is a parallel, open-label, 2-arm prospective, proof-of-concept randomized controlled trial (RCT)"

1b-iv) RESULTS section in abstract must contain use data

Report number of participants enrolled/assessed in each group, the use/uptake of the intervention (e.g., attrition/adherence metrics, use over time, number of logins etc.), in addition to primary/secondary outcomes. (Note: Only report in the abstract what the main paper is reporting. If this information is missing from the main body of text, consider adding it)

	1	2	3	4	5	
subitem not at all important	○	○	○	○	◉	essential

Rensa markering

Does your paper address subitem 1b-iv?

Copy and paste relevant sections from the manuscript abstract (include quotes in quotation marks "like this" to indicate direct quotes from your manuscript), or elaborate on this item by providing additional information not in the ms, or briefly explain why the item is not applicable/relevant for your study

"Twenty-eight participants (14 control, 14 Rehabkompassen®) participated in the current study, with 100 patients screened. The overall recruitment rate was 28%. Retention in the trial was 86% at 12-month follow-up. Meanwhile, all participants used the tool as planned during their follow-ups, which provided 100% task-completion rate of using Rehabkompassen® and suggested excellent feasibility. Both patient (79%)- and physician (100%)-participants reported satisfaction with the instrument. Two physicians (100%) and 18 patients (75%) were willing to use the tool in the future. Furthermore, both modified Rankin Scale (mRS) as the primary outcome and various stroke impacts as secondary outcomes were successfully collected and compared in this study. "

1b-v) CONCLUSIONS/DISCUSSION in abstract for negative trials

Conclusions/Discussions in abstract for negative trials: Discuss the primary outcome - if the trial is negative (primary outcome not changed), and the intervention was not used, discuss whether negative results are attributable to lack of uptake and discuss reasons. (Note: Only report in the abstract what the main paper is reporting. If this information is missing from the main body of text, consider adding it)

	1	2	3	4	5	
subitem not at all important	○	○	○	◉	○	essential

Rensa markering

Does your paper address subitem 1b-v?

Copy and paste relevant sections from the manuscript abstract (include quotes in quotation marks "like this" to indicate direct quotes from your manuscript), or elaborate on this item by providing additional information not in the ms, or briefly explain why the item is not applicable/relevant for your study

"This study demonstrated high feasibility and adherence of the study protocol as well as great acceptability of the Rehabkompassen® among patients with stroke and physicians in an outpatient setting in comparison to the predefined criterion. The information collected in this feasibility study combined with the amendments of the study protocol may improve the future definitive RCT. The results of this trial support the feasibility and acceptability of conducting a large definitive RCT."

INTRODUCTION

2a) In INTRODUCTION: Scientific background and explanation of rationale

2a-i) Problem and the type of system/solution

Describe the problem and the type of system/solution that is object of the study: intended as stand-alone intervention vs. incorporated in broader health care program? Intended for a particular patient population? Goals of the intervention, e.g., being more cost-effective to other interventions, replace or complement other solutions? (Note: Details about the intervention are provided in “Methods” under 5)

	1	2	3	4	5	
subitem not at all important	○	○	○	○	◉	essential

Rensa markering

Does your paper address subitem 2a-i? *

Copy and paste relevant sections from the manuscript (include quotes in quotation marks "like this" to indicate direct quotes from your manuscript), or elaborate on this item by providing additional information not in the ms, or briefly explain why the item is not applicable/relevant for your study

"Before starting a large randomized controlled trial (RCT), recruitment and retention rates, acceptability of the intervention, and adherence to protocol need to be clarified. The aim of this study was to evaluate the feasibility and acceptability of Rehabkompassen® as a digital follow-up tool in the outpatient clinic, in comparison to the control group. "

2a-ii) Scientific background, rationale: What is known about the (type of) system

Scientific background, rationale: What is known about the (type of) system that is the object of the study (be sure to discuss the use of similar systems for other conditions/diagnoses, if appropiate), motivation for the study, i.e. what are the reasons for and what is the context for this specific study, from which stakeholder viewpoint is the study performed, potential impact of findings [2]. Briefly justify the choice of the comparator.

	1	2	3	4	5	
subitem not at all important	○	○	○	○	◉	essential

Rensa markering

Does your paper address subitem 2a-ii? *

Copy and paste relevant sections from the manuscript (include quotes in quotation marks "like this" to indicate direct quotes from your manuscript), or elaborate on this item by providing additional information not in the ms, or briefly explain why the item is not applicable/relevant for your study

"Stroke is the third-leading cause of disability among adults worldwide with heavy burden for the patients and their families as well as society [1, 2]. Recently, a Global Burden of Disease report indicated that there were 143 million disability-adjusted life-years (DALYs) due to stroke globally in 2019 [3]. People with stroke often have heterogenous functional impairments and limitations of various daily and social activities followed by decreased health-related quality of life long after stroke onset. Despite the recommendations by recent Swedish stroke guidelines, structured follow-up to identify patients' rehabilitation needs and provide patient-tailored and precision rehabilitation regimens remains largely lacking in current stroke care [4]. Establishing such care, however, might lead to extra burden for our already time- and resource-constrained healthcare system. Thus, a cost-effective solution is urgently needed to facilitate identifying individual rehabilitation needs and thereby providing patient-tailored rehabilitation to reduce disability among people with stroke.
To meet these challenges, we developed Rehabkompassen®, a novel digital follow-up tool [5], based on well-validated, patient-reported outcome measures (PROMs) [6]. The PROMs used as Rehabkompassen® questionnaires consisted of the simplified modified Rankin Scale questionnaire (smRSq), Fatigue Assessment Scale (FAS), Eating Assessment Tool (EAT-10), Hospital Anxiety and Depression Scale (HADS), Stroke Impact Scale 3.0 plus (SIS+), and EuroQoL 5-dimension 3 levels (EQ-5D-3L) [6]. Rehabkompassen® identifies and graphically visualizes stroke patients' multi-dimensional rehabilitation needs on an individual and group level. The tool can be used as a screening tool for initial triage before the visit, as a communication platform during the visit, and as a support tool for patient referral after the visit. The tool allows serial assessment and may also be used as an evaluation tool after the eventual rehabilitation regimens have been delivered or as an illustration of the alterations of rehabilitation needs over time [5]. Both paper and digital version of the instrument have previously been proven as a feasible, useful, and time-saving tool for identification of unmet rehabilitation needs among persons with stroke [5,7] or transient ischemic attack [8,9] in clinical practice. "

2b) In INTRODUCTION: Specific objectives or hypotheses

Does your paper address CONSORT subitem 2b? *

Copy and paste relevant sections from the manuscript (include quotes in quotation marks "like this" to indicate direct quotes from your manuscript), or elaborate on this item by providing additional information not in the ms, or briefly explain why the item is not applicable/relevant for your study

"The aim of this study was to evaluate the feasibility and acceptability of Rehabkompassen® as a digital follow-up tool in the outpatient clinic, in comparison to the

METHODS

3a) Description of trial design (such as parallel, factorial) including allocation ratio

Does your paper address CONSORT subitem 3a? *

Copy and paste relevant sections from the manuscript (include quotes in quotation marks "like this" to indicate direct quotes from your manuscript), or elaborate on this item by providing additional information not in the ms, or briefly explain why the item is not applicable/relevant for your study

"A parallel, open-label, 2-arm prospective, proof-of-concept pilot RCT with allocation ratio 1:1 was carried out "

3b) Important changes to methods after trial commencement (such as eligibility criteria), with reasons

Does your paper address CONSORT subitem 3b? *

Copy and paste relevant sections from the manuscript (include quotes in quotation marks "like this" to indicate direct quotes from your manuscript), or elaborate on this item by providing additional information not in the ms, or briefly explain why the item is not applicable/relevant for your study

No changes in the current study,
However, "After analysing 3- and 12-months post-visit assessments, we realized that several patient-participants did not fully understand what the Rehabkompassen®graph was despite being satisfied with their outpatient visits with Rehabkompassen®. Therefore, we amended the questions regarding the graph and added a simple picture of a Rehabkompassen®graph to help the participant recall and more easily understand the question. This revised questionnaire will be used in the future definitive RCT."

3b-i) Bug fixes, Downtimes, Content Changes

Bug fixes, Downtimes, Content Changes: ehealth systems are often dynamic systems. A description of changes to methods therefore also includes important changes made on the intervention or comparator during the trial (e.g., major bug fixes or changes in the functionality or content) (5-iii) and other "unexpected events" that may have influenced study design such as staff changes, system failures/downtimes, etc. [2].

	1	2	3	4	5	
subitem not at all important	○	○	○	○	◉	essential

Rensa markering

Does your paper address subitem 3b-i?

Copy and paste relevant sections from the manuscript (include quotes in quotation marks "like this" to indicate direct quotes from your manuscript), or elaborate on this item by providing additional information not in the ms, or briefly explain why the item is not applicable/relevant for your study

"After analysing 3- and 12-months post-visit assessments, we realized that several patient-participants did not fully understand what the Rehabkompassen®graph was despite being satisfied with their outpatient visits with Rehabkompassen®. Therefore, we amended the questions regarding the graph and added a simple picture of a Rehabkompassen®graph to help the participant recall and more easily understand the question. This revised questionnaire will be used in the future definitive RCT."

4a) Eligibility criteria for participants

Does your paper address CONSORT subitem 4a? *

Copy and paste relevant sections from the manuscript (include quotes in quotation marks "like this" to indicate direct quotes from your manuscript), or elaborate on this item by providing additional information not in the ms, or briefly explain why the item is not applicable/relevant for your study

"All patients with a stroke diagnosis during July 2020 – March 2021 were assessed for study eligibility. Inclusion criteria were 1) age > 18 years; 2) a stroke at least 3 months prior to an outpatient visit; and 3) living in the community. Exclusion criteria were 1) inability to answer the evaluation questions; 2) inability to see the Rehabkompassen®graph; and/or 3) lack of a BankID (a Swedish digital authorization tool) since those patients without BankID prior to participating in the study were often digitally naive"

4a-i) Computer / Internet literacy

Computer / Internet literacy is often an implicit “de facto” eligibility criterion - this should be explicitly clarified.

	1	2	3	4	5	
subitem not at all important	○	○	○	○	◉	essential

Rensa markering

Does your paper address subitem 4a-i?

Copy and paste relevant sections from the manuscript (include quotes in quotation marks "like this" to indicate direct quotes from your manuscript), or elaborate on this item by providing additional information not in the ms, or briefly explain why the item is not applicable/relevant for your study

"3) lack of a BankID (a Swedish digital authorization tool) since those patients without BankID prior to participating in the study were often digitally naive"

4a-ii) Open vs. closed, web-based vs. face-to-face assessments:

Open vs. closed, web-based vs. face-to-face assessments: Mention how participants were recruited (online vs. offline), e.g., from an open access website or from a clinic, and clarify if this was a purely web-based trial, or there were face-to-face components (as part of the intervention or for assessment), i.e., to what degree got the study team to know the participant. In online-only trials, clarify if participants were quasi-anonymous and whether having multiple identities was possible or whether technical or logistical measures (e.g., cookies, email confirmation, phone calls) were used to detect/prevent these.

	1	2	3	4	5	
subitem not at all important	○	○	○	○	◉	essential

Rensa markering

Does your paper address subitem 4a-ii? *

Copy and paste relevant sections from the manuscript (include quotes in quotation marks "like this" to indicate direct quotes from your manuscript), or elaborate on this item by providing additional information not in the ms, or briefly explain why the item is not applicable/relevant for your study

"All patients with a stroke diagnosis during July 2020 – March 2021 were assessed for study eligibility" "All patients who met the inclusion criteria, together with the appointment for as usual outpatient follow-up, received an invitation to the study around 2 months after stroke onset (Figure 1). Patients who gave their consent were contacted via telephone by a research staff person at the clinic to provide oral information about the study. Written consent was obtained from all participants before participation in the study. "

4a-iii) Information giving during recruitment

Information given during recruitment. Specify how participants were briefed for recruitment and in the informed consent procedures (e.g., publish the informed consent documentation as appendix, see also item X26), as this information may have an effect on user self-selection, user expectation and may also bias results.

	1	2	3	4	5	
subitem not at all important	○	○	○	○	●	essential

Rensa markering

Does your paper address subitem 4a-iii?

Copy and paste relevant sections from the manuscript (include quotes in quotation marks "like this" to indicate direct quotes from your manuscript), or elaborate on this item by providing additional information not in the ms, or briefly explain why the item is not applicable/relevant for your study

"All patients who met the inclusion criteria, together with the appointment for as usual outpatient follow-up, received an invitation to the study around 2 months after stroke onset (Figure 1). Patients who gave their consent were contacted via telephone by a research staff person at the clinic to provide oral information about the study. Written consent was obtained from all participants before participation in the study. "

4b) Settings and locations where the data were collected

Does your paper address CONSORT subitem 4b? *

Copy and paste relevant sections from the manuscript (include quotes in quotation marks "like this" to indicate direct quotes from your manuscript), or elaborate on this item by providing additional information not in the ms, or briefly explain why the item is not applicable/relevant for your study

" All patients who met the inclusion criteria, together with the appointment for as usual outpatient follow-up, received an invitation to the study around 2 months after stroke onset "

4b-i) Report if outcomes were (self-)assessed through online questionnaires

Clearly report if outcomes were (self-)assessed through online questionnaires (as common in web-based trials) or otherwise.

	1	2	3	4	5	
subitem not at all important	○	○	○	○	◉	essential

Rensa markering

Does your paper address subitem 4b-i? *

Copy and paste relevant sections from the manuscript (include quotes in quotation marks "like this" to indicate direct quotes from your manuscript), or elaborate on this item by providing additional information not in the ms, or briefly explain why the item is not applicable/relevant for your study

"Acceptability was assessed in terms of the patient-participants' satisfaction with Rehabkompassen®. After the 3- and 12-month follow-up visits, all patient-participants in both intervention and control groups answered a satisfaction questionnaire through 1177.se. The questionnaire addressed their overall experiences of the conversation with the physician and their satisfaction of using the Rehabkompassen®graph during the follow-up visit. The satisfaction of Rehabkompassen® was rated in terms of how it affected their ability to understand their rehabilitation needs during the consultation throughout the outpatient visit by using a Likert scale from 1 (very easy) to 5 (very difficult). Participants rating either very easy or fairly easy were considered as satisfied with the tool. The patients' -satisfaction rate was calculated by the number of patients who were satisfied with the tool divided by the total number of patient-participants."

4b-ii) Report how institutional affiliations are displayed

Report how institutional affiliations are displayed to potential participants [on ehealth media], as affiliations with prestigious hospitals or universities may affect volunteer rates, use, and reactions with regards to an intervention.(Not a required item – describe only if this may bias results)

	1	2	3	4	5	
subitem not at all important	○	○	●	○	○	essential

Rensa markering

Does your paper address subitem 4b-ii?

Copy and paste relevant sections from the manuscript (include quotes in quotation marks "like this" to indicate direct quotes from your manuscript), or elaborate on this item by providing additional information not in the ms, or briefly explain why the item is not applicable/relevant for your study

"A parallel, open-label, 2-arm prospective, proof-of-concept pilot RCT with allocation ratio 1:1 was carried out in an outpatient clinical setting in the Department of Neurological Rehabilitation, University Hospital of Umeå, Sweden"

5) The interventions for each group with sufficient details to allow replication, including how and when they were actually administered

5-i) Mention names, credential, affiliations of the developers, sponsors, and owners

Mention names, credential, affiliations of the developers, sponsors, and owners [6] (if authors/evaluators are owners or developer of the software, this needs to be declared in a “Conflict of interest” section or mentioned elsewhere in the manuscript).

	1	2	3	4	5	
subitem not at all important	○	○	○	○	◉	essential

Rensa markering

Does your paper address subitem 5-i?

Copy and paste relevant sections from the manuscript (include quotes in quotation marks "like this" to indicate direct quotes from your manuscript), or elaborate on this item by providing additional information not in the ms, or briefly explain why the item is not applicable/relevant for your study

"At 3-month Follow-up
The intervention group consisted of the Rehabkompassen® and as-usual outpatient follow-up that included patient's history of disease, examination, and rehabilitation treatment plan. Around 2 months after stroke onset, the patient-participants in the intervention group received the Rehabkompassen® questionnaires in their inbox at 1177.se, which is a Swedish-government-issued digital platform for citizens' healthcare as described in the previous study [5]. The patient-participants filled in the Rehabkompassen® questionnaires [7] at home by clicking on the links in their e-mail inbox at 1177.se. The Rehabkompassen® questionnaires had to be answered no later than one week prior to the 3-month follow-up visit (Figure 1).
Prior to the 3-month follow-up, a nurse prioritized the team recourse based on the patient's Rehabkompassen®. During the follow-up visit, a doctor showed the patient's personal Rehabkompassen®graph (Figure 1A as an example at 3-month follow-up) on the computer and used it as an illustration to discuss with patients their health status and rehabilitation needs.
The control group received as-usual follow-up without Rehabkompassen® with otherwise identical procedures as the intervention group. To collect the baseline data, the control group filled in only two questionnaires (smRSq and EQ-5D-3L) via 1177.se prior to their follow-up appointments (Figure 1).
The length of each visit in both intervention and control groups was the same, approximately 45 minutes. After the visit, all participants in both intervention and control groups received various rehabilitation regimens based on their rehabilitation needs.
At 12-month Follow-up
All participants from the control and intervention groups filled in the Rehabkompassen® questionnaires via 1177.se at home one week prior a 12-month follow-up visit (Figure 1). The patients' Rehabkompassen®graphs were utilized in combination with the usual outpatient follow-up as described above for the intervention group. The Rehabkompassen®graph (Figure 2A as an example at 3-month follow-up for those in the intervention group) could be used as an evaluation tool to compare with the Rehabkompassen®graph at 12-month follow-up (Figure 2B)."

5-ii) Describe the history/development process

Describe the history/development process of the application and previous formative evaluations (e.g., focus groups, usability testing), as these will have an impact on adoption/use rates and help with interpreting results.

	1	2	3	4	5	
subitem not at all important	○	○	●	○	○	essential

Rensa markering

Does your paper address subitem 5-ii?

Copy and paste relevant sections from the manuscript (include quotes in quotation marks "like this" to indicate direct quotes from your manuscript), or elaborate on this item by providing additional information not in the ms, or briefly explain why the item is not applicable/relevant for your study

"To meet these challenges, we developed Rehabkompassen®, a novel digital follow-up tool [5], based on well-validated, patient-reported outcome measures (PROMs) [6]. The PROMs used as Rehabkompassen® questionnaires consisted of the simplified modified Rankin Scale questionnaire (smRSq), Fatigue Assessment Scale (FAS), Eating Assessment Tool (EAT-10), Hospital Anxiety and Depression Scale (HADS), Stroke Impact Scale 3.0 plus (SIS+), and EuroQoL 5-dimension 3 levels (EQ-5D-3L) [6]. Rehabkompassen® identifies and graphically visualizes stroke patients' multi-dimensional rehabilitation needs on an individual and group level. The tool can be used as a screening tool for initial triage before the visit, as a communication platform during the visit, and as a support tool for patient referral after the visit. The tool allows serial assessment and may also be used as an evaluation tool after the eventual rehabilitation regimens have been delivered or as an illustration of the alterations of rehabilitation needs over time [5]. Both paper and digital version of the instrument have previously been proven as a feasible, useful, and time-saving tool for identification of unmet rehabilitation needs among persons with stroke [5,7] or transient ischemic attack [8,9] in clinical practice."

5-iii) Revisions and updating

Revisions and updating. Clearly mention the date and/or version number of the application/intervention (and comparator, if applicable) evaluated, or describe whether the intervention underwent major changes during the evaluation process, or whether the development and/or content was "frozen" during the trial. Describe dynamic components such as news feeds or changing content which may have an impact on the replicability of the intervention (for unexpected events see item 3b).

	1	2	3	4	5	
subitem not at all important	○	○	○	○	◉	essential

Rensa markering

Does your paper address subitem 5-iii?

Copy and paste relevant sections from the manuscript (include quotes in quotation marks "like this" to indicate direct quotes from your manuscript), or elaborate on this item by providing additional information not in the ms, or briefly explain why the item is not applicable/relevant for your study

"After analysing 3- and 12-months post-visit assessments, we realized that several patient-participants did not fully understand what the Rehabkompassen®graph was despite being satisfied with their outpatient visits with Rehabkompassen®. Therefore, we amended the questions regarding the graph and added a simple picture of a Rehabkompassen®graph to help the participant recall and more easily understand the question. This revised questionnaire will be used in the future definitive RCT."

5-iv) Quality assurance methods

Provide information on quality assurance methods to ensure accuracy and quality of information provided [1], if applicable.

	1	2	3	4	5	
subitem not at all important	○	○	○	○	◉	essential

Rensa markering

Does your paper address subitem 5-iv?

Copy and paste relevant sections from the manuscript (include quotes in quotation marks "like this" to indicate direct quotes from your manuscript), or elaborate on this item by providing additional information not in the ms, or briefly explain why the item is not applicable/relevant for your study

"The length of each visit in both intervention and control groups was the same, approximately 45 minutes. After the visit, all participants in both intervention and control groups received various rehabilitation regimens based on their rehabilitation needs."

5-v) Ensure replicability by publishing the source code, and/or providing screenshots/screen-capture video, and/or providing flowcharts of the algorithms used

Ensure replicability by publishing the source code, and/or providing screenshots/screen-capture video, and/or providing flowcharts of the algorithms used. Replicability (i.e., other researchers should in principle be able to replicate the study) is a hallmark of scientific reporting.

	1	2	3	4	5	
subitem not at all important	○	○	○	○	◉	essential

Rensa markering

Does your paper address subitem 5-v?

Copy and paste relevant sections from the manuscript (include quotes in quotation marks "like this" to indicate direct quotes from your manuscript), or elaborate on this item by providing additional information not in the ms, or briefly explain why the item is not applicable/relevant for your study

pleases see Figure 1. "All participants from the control and intervention groups filled in the Rehabkompassen® questionnaires via 1177.se at home one week prior a 12-month follow-up visit (Figure 1). "

5-vi) Digital preservation

Digital preservation: Provide the URL of the application, but as the intervention is likely to change or disappear over the course of the years; also make sure the intervention is archived (Internet Archive, webcitation.org, and/or publishing the source code or screenshots/videos alongside the article). As pages behind login screens cannot be archived, consider creating demo pages which are accessible without login.

	1	2	3	4	5	
subitem not at all important	◉	○	○	○	○	essential

Rensa markering

Does your paper address subitem 5-vi?

Copy and paste relevant sections from the manuscript (include quotes in quotation marks "like this" to indicate direct quotes from your manuscript), or elaborate on this item by providing additional information not in the ms, or briefly explain why the item is not applicable/relevant for your study

The Rehabkompassen® is not web-based; and thus has not URL

5-vii) Access

Access: Describe how participants accessed the application, in what setting/context, if they had to pay (or were paid) or not, whether they had to be a member of specific group. If known, describe how participants obtained “access to the platform and Internet” [1]. To ensure access for editors/reviewers/readers, consider to provide a “backdoor” login account or demo mode for reviewers/readers to explore the application (also important for archiving purposes, see vi).

	1	2	3	4	5	
subitem not at all important	◉	○	○	○	○	essential

Rensa markering

Does your paper address subitem 5-vii? *

Copy and paste relevant sections from the manuscript (include quotes in quotation marks "like this" to indicate direct quotes from your manuscript), or elaborate on this item by providing additional information not in the ms, or briefly explain why the item is not applicable/relevant for your study

This item is not relevant

5-viii) Mode of delivery, features/functionalities/components of the intervention and comparator, and the theoretical framework

Describe mode of delivery, features/functionalities/components of the intervention and comparator, and the theoretical framework [6] used to design them (instructional strategy [1], behaviour change techniques, persuasive features, etc., see e.g., [7, 8] for terminology). This includes an in-depth description of the content (including where it is coming from and who developed it) [1],” whether [and how] it is tailored to individual circumstances and allows users to track their progress and receive feedback” [6]. This also includes a description of communication delivery channels and – if computer-mediated communication is a component – whether communication was synchronous or asynchronous [6]. It also includes information on presentation strategies [1], including page design principles, average amount of text on pages, presence of hyperlinks to other resources, etc. [1].

	1	2	3	4	5	
subitem not at all important	○	○	○	○	◉	essential

Rensa markering

Does your paper address subitem 5-viii? *

Copy and paste relevant sections from the manuscript (include quotes in quotation marks "like this" to indicate direct quotes from your manuscript), or elaborate on this item by providing additional information not in the ms, or briefly explain why the item is not applicable/relevant for your study

"The intervention group consisted of the Rehabkompassen® and as-usual outpatient follow-up that included patient's history of disease, examination, and rehabilitation treatment plan. Around 2 months after stroke onset, the patient-participants in the intervention group received the Rehabkompassen® questionnaires in their inbox at 1177.se, which is a Swedish-government-issued digital platform for citizens' healthcare as described in the previous study [5]. The patient-participants filled in the Rehabkompassen® questionnaires [7] at home by clicking on the links in their e-mail inbox at 1177.se. The Rehabkompassen® questionnaires had to be answered no later than one week prior to the 3-month follow-up visit (Figure 1).

Prior to the 3-month follow-up, a nurse prioritized the team recourse based on the patient's Rehabkompassen®. During the follow-up visit, a doctor showed the patient's personal Rehabkompassen®graph (Figure 1A as an example at 3-month follow-up) on the computer and used it as an illustration to discuss with patients their health status and rehabilitation needs. "

5-ix) Describe use parameters

Describe use parameters (e.g., intended "doses" and optimal timing for use). Clarify what instructions or recommendations were given to the user, e.g., regarding timing, frequency, heaviness of use, if any, or was the intervention used ad libitum.

	1	2	3	4	5	
subitem not at all important	○	○	○	○	◉	essential

Rensa markering

Does your paper address subitem 5-ix?

Copy and paste relevant sections from the manuscript (include quotes in quotation marks "like this" to indicate direct quotes from your manuscript), or elaborate on this item by providing additional information not in the ms, or briefly explain why the item is not applicable/relevant for your study

"Prior to the 3-month follow-up, a nurse prioritized the team recourse based on the patient's Rehabkompassen®. During the follow-up visit, a doctor showed the patient's personal " "All participants from the control and intervention groups filled in the Rehabkompassen® questionnaires via 1177.se at home one week prior a 12-month follow-up visit "

5-x) Clarify the level of human involvement

Clarify the level of human involvement (care providers or health professionals, also technical assistance) in the e-intervention or as co-intervention (detail number and expertise of professionals involved, if any, as well as "type of assistance offered, the timing and frequency of the support, how it is initiated, and the medium by which the assistance is delivered". It may be necessary to distinguish between the level of human involvement required for the trial, and the level of human involvement required for a routine application outside of a RCT setting (discuss under item 21 – generalizability).

	1	2	3	4	5	
subitem not at all important	○	○	○	○	◉	essential

Rensa markering

Does your paper address subitem 5-x?

Copy and paste relevant sections from the manuscript (include quotes in quotation marks "like this" to indicate direct quotes from your manuscript), or elaborate on this item by providing additional information not in the ms, or briefly explain why the item is not applicable/relevant for your study

"The patient-participants filled in the Rehabkompassen® questionnaires [7] at home by clicking on the links in their e-mail inbox at 1177.se. The Rehabkompassen® questionnaires had to be answered no later than one week prior to the 3-month follow-up visit (Figure 1). Prior to the 3-month follow-up, a nurse prioritized the team recourse based on the patient's Rehabkompassen®. During the follow-up visit, a doctor showed the patient's personal Rehabkompassen®graph (Figure 1A as an example at 3-month follow-up) on the computer and used it as an illustration to discuss with patients their health status and rehabilitation needs. "

5-xi) Report any prompts/reminders used

Report any prompts/reminders used: Clarify if there were prompts (letters, emails, phone calls, SMS) to use the application, what triggered them, frequency etc. It may be necessary to distinguish between the level of prompts/reminders required for the trial, and the level of prompts/reminders for a routine application outside of a RCT setting (discuss under item 21 – generalizability).

	1	2	3	4	5	
subitem not at all important	○	○	○	●	○	essential

Rensa markering

Does your paper address subitem 5-xi? *

Copy and paste relevant sections from the manuscript (include quotes in quotation marks "like this" to indicate direct quotes from your manuscript), or elaborate on this item by providing additional information not in the ms, or briefly explain why the item is not applicable/relevant for your study

Patients will receive reminders via a telephone call by a research staff if they are not filled in the questionnaires. The detail presented in the reference No. 5 in the references.

5-xii) Describe any co-interventions (incl. training/support)

Describe any co-interventions (incl. training/support): Clearly state any interventions that are provided in addition to the targeted eHealth intervention, as ehealth intervention may not be designed as stand-alone intervention. This includes training sessions and support [1]. It may be necessary to distinguish between the level of training required for the trial, and the level of training for a routine application outside of a RCT setting (discuss under item 21 – generalizability.

	1	2	3	4	5	
subitem not at all important	○	○	○	○	◉	essential

Rensa markering

Does your paper address subitem 5-xii? *

Copy and paste relevant sections from the manuscript (include quotes in quotation marks "like this" to indicate direct quotes from your manuscript), or elaborate on this item by providing additional information not in the ms, or briefly explain why the item is not applicable/relevant for your study

" After the visit, all participants in both intervention and control groups received various rehabilitation regimens based on their rehabilitation needs"

6a) Completely defined pre-specified primary and secondary outcome measures, including how and when they were assessed

Does your paper address CONSORT subitem 6a? *

Copy and paste relevant sections from the manuscript (include quotes in quotation marks "like this" to indicate direct quotes from your manuscript), or elaborate on this item by providing additional information not in the ms, or briefly explain why the item is not applicable/relevant for your study

" To study the feasibility of the study, the information on recruitment rate, adherence, delivery and uptake of the Rehabkompassen®, satisfaction, and possible future use were collected in this study [11,12]. We predefined the following thresholds for specific feasibility and acceptability criteria for deciding whether to progress to the next stage (i.e., to carry out the future definitive RCT): 1) the patient recruitment rate would be 20% or greater of the total number of patients who were asked to participate in the study; 2) adherence of study protocol would be more than 60% of the total number of the participants with a written consent; 3) the feasibility (delivery/usage) of Rehabkompassen® would be more than 60% of the total number of patients using the Rehabkompassen® as planned; 4) the acceptability of Rehabkompassen® (the mean level of satisfaction from both patients and physicians) would be 60% or greater of the total participants; and 5) willingness to use the tool in the future would be more than 60% of the total participants (Table 1). However, not reaching the pre-defined criteria does not necessarily indicate unfeasibility of the trial but rather underlines that some changes to the protocol are needed.
Primary Outcome
The smRSq [13-15] is a questionnaire used to collect a primary outcome of the modified Rankin Scale (mRS) that measure patients' independence/disability level in their daily activities in the future definitive RCT. The smRSq is based on the yes/no responses to five questions, which in turn can be used to calculate the mRS 0-5 [13]. A favorable outcome will be defined as mRS score of 0-2 (no symptoms to independent but with minor disability). A poor outcome will be defined as mRS score of 3-5 (disability but able to walk to bed-bound and in need of full nursing care) or 6 (death). The completion rates, variances, and 95% confidence intervals for the difference between the intervention arms will be analyzed.
Secondary Outcomes
Secondary outcomes were assessed and collected directly after patients filled in the Rehabkompassen® questionnaires.
Fatigue is measured by FAS [16], a questionnaire used for identifying symptoms of chronic fatigue. It is comprised of 10 questions regarding both physical and mental fatigue answered on a scale from 1 (never) to 5 (always).
Dysphagia is assessed by EAT-10 [17] including 10 questions concerning swallowing difficulties. Each question is to be answered on a scale from 0 (no problem) to 4 (severe problem).

Depression and Anxiety are measured by HADS [18], a screening tool for assessment of anxiety and depression. It comprises seven questions about anxiety and seven questions about depression answered on a scale from 0 (no symptoms) to 3 (severe symptoms). The subscales for anxiety and depression are added and interpreted separately.
Stroke impacts are assessed by SIS [19], a patient-reported, stroke-specific outcome measurement containing 59 questions and a visual analog scale (VAS) for estimation of perceived stroke recovery. As secondary outcomes, the proposed study assesses stroke impacts within 8 domains, namely strength, memory/cognition, feelings/emotions, communication, personal activities of daily living (ADLs), instrumental ADLs, mobility, motor impact, and social participation. In the previous study, we also added items covering continence and sexual function as well as sleep disturbance, which was named SIS-plus (SIS+)[7]. The SIS data present in ordinal score ranges from 0–100, with higher scores indicating less impact of stroke [19].
Health-related quality of life and cost-effectiveness were measured by EQ-5D-3L [20, 21]. EQ-5D-3L consists of two parts: a VAS and a descriptive system covering five dimensions of health (mobility, hygiene, usual activities, pain/discomfort, and anxiety/depression) with three response alternatives (ranging from no problems to extreme problems). The latter can be translated to an index value with anchor points 0 (death) and 1 (full health) for eliciting the overall health utility score, corresponding to a quality-adjusted life years (QALYs) score.
"

6a-i) Online questionnaires: describe if they were validated for online use and apply CHERRIES items to describe how the questionnaires were designed/deployed

If outcomes were obtained through online questionnaires, describe if they were validated for online use and apply CHERRIES items to describe how the questionnaires were designed/deployed [9].

	1	2	3	4	5	
subitem not at all important	◉	○	○	○	○	essential

Rensa markering

Does your paper address subitem 6a-i?

Copy and paste relevant sections from manuscript text

No, the questionnaires used in the current study are not on-line.

6a-ii) Describe whether and how “use” (including intensity of use/dosage) was defined/measured/monitored

Describe whether and how “use” (including intensity of use/dosage) was defined/measured/monitored (logins, logfile analysis, etc.). Use/adoption metrics are important process outcomes that should be reported in any ehealth trial.

	1	2	3	4	5	
subitem not at all important	○	○	○	○	◉	essential

Rensa markering

Does your paper address subitem 6a-ii?

Copy and paste relevant sections from manuscript text

They used at 3 and 12-month follow-up .
"At 3-month Follow-up
The intervention group consisted of the Rehabkompassen® and as-usual outpatient follow-up that included patient’s history of disease, examination, and rehabilitation treatment plan. Around 2 months after stroke onset, the patient-participants in the intervention group received the Rehabkompassen® questionnaires in their inbox at 1177.se, which is a Swedish-government-issued digital platform for citizens’ healthcare as described in the previous study "
"At 12-month Follow-up
All participants from the control and intervention groups filled in the Rehabkompassen® questionnaires via 1177.se at home one week prior a 12-month follow-up visit "

6a-iii) Describe whether, how, and when qualitative feedback from participants was obtained

Describe whether, how, and when qualitative feedback from participants was obtained (e.g., through emails, feedback forms, interviews, focus groups).

	1	2	3	4	5	
subitem not at all important	○	○	○	○	◉	essential

Rensa markering

Does your paper address subitem 6a-iii?

Copy and paste relevant sections from manuscript text

"Post-visit Assessments of Satisfaction with the Rehabkompassen®
Acceptability was assessed in terms of the patient-participants' satisfaction with Rehabkompassen®. After the 3- and 12-month follow-up visits, all patient-participants in both intervention and control groups answered a satisfaction questionnaire through 1177.se. The questionnaire addressed their overall experiences of the conversation with the physician and their satisfaction of using the Rehabkompassen®graph during the follow-up visit. The satisfaction of Rehabkompassen® was rated in terms of how it affected their ability to understand their rehabilitation needs during the consultation throughout the outpatient visit by using a Likert scale from 1 (very easy) to 5 (very difficult). Participants rating either very easy or fairly easy were considered as satisfied with the tool. The patients' -satisfaction rate was calculated by the number of patients who were satisfied with the tool divided by the total number of patient-participants.
In the end of the 3-month follow-ups, two physicians involved in the study answered a questionnaire with 5 questions regarding different aspects of utility for providing feedback on perceived feasibility and satisfaction of the instrument in clinical practice. A Likert scale ranging from 1 (strongly disagree) to 5 (strongly agree) was used with higher scores indicating better outcomes. Ratings of strongly agree and fairly agree were considered as satisfied with the tool. The physicians' satisfaction rate was calculated by the total number of satisfied aspects divided by the total number of aspects.
After analysing 3- and 12-months post-visit assessments, we realized that several patient-participants did not fully understand what the Rehabkompassen®graph was despite being satisfied with their outpatient visits with Rehabkompassen®. Therefore, we amended the questions regarding the graph and added a simple picture of a Rehabkompassen®graph to help the participant recall and more easily understand the question. This revised questionnaire will be used in the future definitive RCT."

6b) Any changes to trial outcomes after the trial commenced, with reasons

Does your paper address CONSORT subitem 6b? *

Copy and paste relevant sections from the manuscript (include quotes in quotation marks "like this" to indicate direct quotes from your manuscript), or elaborate on this item by providing additional information not in the ms, or briefly explain why the item is not applicable/relevant for your study

"Although no statistically significant difference on mRS as the primary outcome is expected to be found in this feasibility study, this still raised a critical concern on whether the mRS as a single primary outcome was sensitive enough to capture the subtle alterations of treatment-effects in the future definitive RCT. Meanwhile, the background characteristics of the participants demonstrated most of the target study population had a mild to moderate disability with more limitation on social participation, which is in line with previous Swedish stroke RCTs [14,23]. In order to catch the minor but important changes on both daily activity and social participation over time, we added the Domain 8 in SIS (SIS-D8) [24] as another primary outcome to use in the future definitive RCT, since mRS covers mainly daily activity [15]. "

7a) How sample size was determined

NPT: When applicable, details of whether and how the clustering by care provides or centers was addressed

7a-i) Describe whether and how expected attrition was taken into account when calculating the sample size

Describe whether and how expected attrition was taken into account when calculating the sample size.

	1	2	3	4	5	
subitem not at all important	◉	○	○	○	○	essential

Rensa markering

Does your paper address subitem 7a-i?

Copy and paste relevant sections from manuscript title (include quotes in quotation marks "like this" to indicate direct quotes from your manuscript), or elaborate on this item by providing additional information not in the ms, or briefly explain why the item is not applicable/relevant for your study

No sample size calculation is need in the current study since it is a feasibility study.
"At this stage, the results remain hardly to be generalized due to its limited sample size (two physicians, 14 patients in contro- and intervention groups respectively) in this pilot study, a further definitive RCT is thus needed."

7b) When applicable, explanation of any interim analyses and stopping guidelines

Does your paper address CONSORT subitem 7b? *

Copy and paste relevant sections from the manuscript (include quotes in quotation marks "like this" to indicate direct quotes from your manuscript), or elaborate on this item by providing additional information not in the ms, or briefly explain why the item is not applicable/relevant for your study

No, It is not relevant since the current study is a feasibility study only.

8a) Method used to generate the random allocation sequence

NPT: When applicable, how care providers were allocated to each trial group

Does your paper address CONSORT subitem 8a? *

Copy and paste relevant sections from the manuscript (include quotes in quotation marks "like this" to indicate direct quotes from your manuscript), or elaborate on this item by providing additional information not in the ms, or briefly explain why the item is not applicable/relevant for your study

"A parallel, open-label, 2-arm prospective, proof-of-concept pilot RCT with allocation ratio 1:1 was carried out in an outpatient clinical setting "

8b) Type of randomisation; details of any restriction (such as blocking and block size)

Does your paper address CONSORT subitem 8b? *

Copy and paste relevant sections from the manuscript (include quotes in quotation marks "like this" to indicate direct quotes from your manuscript), or elaborate on this item by providing additional information not in the ms, or briefly explain why the item is not applicable/relevant for your study

"A parallel, open-label, 2-arm prospective, proof-of-concept pilot RCT with allocation ratio 1:1 was carried out in an outpatient clinical setting "

9) Mechanism used to implement the random allocation sequence (such as sequentially numbered containers), describing any steps taken to conceal the sequence until interventions were assigned

Does your paper address CONSORT subitem 9? *

Copy and paste relevant sections from the manuscript (include quotes in quotation marks "like this" to indicate direct quotes from your manuscript), or elaborate on this item by providing additional information not in the ms, or briefly explain why the item is not applicable/relevant for your study

"Patients who gave their consent were contacted via telephone by a research staff person at the clinic to provide oral information about the study and receive their randomized information"

10) Who generated the random allocation sequence, who enrolled participants, and who assigned participants to interventions

Does your paper address CONSORT subitem 10? *

Copy and paste relevant sections from the manuscript (include quotes in quotation marks "like this" to indicate direct quotes from your manuscript), or elaborate on this item by providing additional information not in the ms, or briefly explain why the item is not applicable/relevant for your study

"The randomization list was created by an independent statistician, who was not involved in outcome assessment or the patient’s treatment. "

11a) If done, who was blinded after assignment to interventions (for example, participants, care providers, those assessing outcomes) and how

NPT: Whether or not administering co-interventions were blinded to group assignment

11a-i) Specify who was blinded, and who wasn't

Specify who was blinded, and who wasn't. Usually, in web-based trials it is not possible to blind the participants [1, 3] (this should be clearly acknowledged), but it may be possible to blind outcome assessors, those doing data analysis or those administering co-interventions (if any).

	1	2	3	4	5	
subitem not at all important	○	○	○	○	◉	essential

Rensa markering

Does your paper address subitem 11a-i? *

Copy and paste relevant sections from the manuscript (include quotes in quotation marks "like this" to indicate direct quotes from your manuscript), or elaborate on this item by providing additional information not in the ms, or briefly explain why the item is not applicable/relevant for your study

Since it is a open-label study, neither patients nor research staff were blinded.
"A parallel, open-label, 2-arm prospective, proof-of-concept pilot RCT with allocation ratio 1:1 was carried out in an outpatient clinical setting "

11a-ii) Discuss e.g., whether participants knew which intervention was the "intervention of interest" and which one was the "comparator"

Informed consent procedures (4a-ii) can create biases and certain expectations - discuss e.g., whether participants knew which intervention was the "intervention of interest" and which one was the "comparator".

	1	2	3	4	5	
subitem not at all important	○	○	○	○	◉	essential

Rensa markering

Does your paper address subitem 11a-ii?

Copy and paste relevant sections from the manuscript (include quotes in quotation marks "like this" to indicate direct quotes from your manuscript), or elaborate on this item by providing additional information not in the ms, or briefly explain why the item is not applicable/relevant for your study

"Patients who gave their consent were contacted via telephone by a research staff person at the clinic to provide oral information about the study and receive their randomized information. "

11b) If relevant, description of the similarity of interventions

(this item is usually not relevant for ehealth trials as it refers to similarity of a placebo or sham intervention to a active medication/intervention)

Does your paper address CONSORT subitem 11b? *

Copy and paste relevant sections from the manuscript (include quotes in quotation marks "like this" to indicate direct quotes from your manuscript), or elaborate on this item by providing additional information not in the ms, or briefly explain why the item is not applicable/relevant for your study

"The control group received as-usual follow-up without Rehabkompassen® with otherwise identical procedures as the intervention group"

12a) Statistical methods used to compare groups for primary and secondary outcomes

NPT: When applicable, details of whether and how the clustering by care providers or centers was addressed

Does your paper address CONSORT subitem 12a? *

Copy and paste relevant sections from the manuscript (include quotes in quotation marks "like this" to indicate direct quotes from your manuscript), or elaborate on this item by providing additional information not in the ms, or briefly explain why the item is not applicable/relevant for your study

"Data Presentations and Statistics
Descriptive statistics were presented with mean and median values, standard deviation, quartiles and proportions. The recruitment rate was calculated by the number of the participants in each group devided by the total number of patients who were assessed for eligibility. The other remaining rate (%) was calculated by the number of the participants in each criterion divided by the number of the patient recuiated in its group. In the Rehabkompassen®graph, PROMs scales data were converted to a scale from 0 (worst outcome) to 100 (best outcome) but unchanged in terms of variable properties [6,7]. Although no statistically significant difference is expected to be found in this feasibility study, the differences on the primary and secondary outcomes on ordinal scale at the 12-month follow-up between intervention and control groups were tested using ordinal logistic regression.
All data were analyzed using the Statistical Package for the Social Sciences (SPSS) version 26.0 Software for Windows (SPSS, Chicago, IL, USA). The figures were generated by GraphPad Prism 9 (San Diego, CA, USA). A two-tailed P-value < 0.05 was considered significant. "

12a-i) Imputation techniques to deal with attrition / missing values

Imputation techniques to deal with attrition / missing values: Not all participants will use the intervention/comparator as intended and attrition is typically high in ehealth trials. Specify how participants who did not use the application or dropped out from the trial were treated in the statistical analysis (a complete case analysis is strongly discouraged, and simple imputation techniques such as LOCF may also be problematic [4]).

	1	2	3	4	5	
subitem not at all important	○	◉	○	○	○	essential

Rensa markering

Does your paper address subitem 12a-i? *

Copy and paste relevant sections from the manuscript (include quotes in quotation marks "like this" to indicate direct quotes from your manuscript), or elaborate on this item by providing additional information not in the ms, or briefly explain why the item is not applicable/relevant for your study

No, since this is only a feasibility study.

12b) Methods for additional analyses, such as subgroup analyses and adjusted analyses

Does your paper address CONSORT subitem 12b? *

Copy and paste relevant sections from the manuscript (include quotes in quotation marks "like this" to indicate direct quotes from your manuscript), or elaborate on this item by providing additional information not in the ms, or briefly explain why the item is not applicable/relevant for your study

No, since this is only a feasibility study.

X26) REB/IRB Approval and Ethical Considerations [recommended as subheading under "Methods"] (not a CONSORT item)

X26-i) Comment on ethics committee approval

	1	2	3	4	5	
subitem not at all important	○	○	○	○	◉	essential

Rensa markering

Does your paper address subitem X26-i?

Copy and paste relevant sections from the manuscript (include quotes in quotation marks "like this" to indicate direct quotes from your manuscript), or elaborate on this item by providing additional information not in the ms, or briefly explain why the item is not applicable/relevant for your study

"Ethical approvals were obtained from the regional Ethical Review Board in Umeå, Sweden with Dnr 2015 / 144-31; and completed with Dnr 2019-02830. "

x26-ii) Outline informed consent procedures

Outline informed consent procedures e.g., if consent was obtained offline or online (how? Checkbox, etc.?), and what information was provided (see 4a-ii). See [6] for some items to be included in informed consent documents.

	1	2	3	4	5	
subitem not at all important	○	○	○	○	◉	essential

Rensa markering

Does your paper address subitem X26-ii?

Copy and paste relevant sections from the manuscript (include quotes in quotation marks "like this" to indicate direct quotes from your manuscript), or elaborate on this item by providing additional information not in the ms, or briefly explain why the item is not applicable/relevant for your study

"Patients who gave their consent were contacted via telephone by a research staff person at the clinic to provide oral information about the study and receive their randomized information. Written consent was obtained from all participants before participation in the study. "

X26-iii) Safety and security procedures

Safety and security procedures, incl. privacy considerations, and any steps taken to reduce the likelihood or detection of harm (e.g., education and training, availability of a hotline)

	1	2	3	4	5	
subitem not at all important	○	◉	○	○	○	essential

Rensa markering

Does your paper address subitem X26-iii?

Copy and paste relevant sections from the manuscript (include quotes in quotation marks "like this" to indicate direct quotes from your manuscript), or elaborate on this item by providing additional information not in the ms, or briefly explain why the item is not applicable/relevant for your study

This question has been adredressed in our previous study (reference no 5).

RESULTS

13a) For each group, the numbers of participants who were randomly assigned, received intended treatment, and were analysed for the primary outcome

NPT: The number of care providers or centers performing the intervention in each group and the number of patients treated by each care provider in each center

Does your paper address CONSORT subitem 13a? *

Copy and paste relevant sections from the manuscript (include quotes in quotation marks "like this" to indicate direct quotes from your manuscript), or elaborate on this item by providing additional information not in the ms, or briefly explain why the item is not applicable/relevant for your study

"One hundred patients were assessed for eligibility (Figure 3) from July 2020 to March 2021, to a high extent coinciding with the second wave of COVID-19 in Sweden. Twenty-eight participants gave written consent, which equated to a recruitment rate of 28%. Among the 72 patients who did not participate in the study (Figure 3), 50 patients never responded to the study invitation letter; 4 patients did not meet inclusion criteria; six patients declined without giving a reason; and one patient died. The remaining 11 patients (11%) declined participation due to various technical issues, such as no computer at home, no Internet, no BankID, and/or inability to use these technologies."

13b) For each group, losses and exclusions after randomisation, together with reasons

Does your paper address CONSORT subitem 13b? (NOTE: Preferably, this is shown in a CONSORT flow diagram) *

Copy and paste relevant sections from the manuscript (include quotes in quotation marks "like this" to indicate direct quotes from your manuscript), or elaborate on this item by providing additional information not in the ms, or briefly explain why the item is not applicable/relevant for your study

"Four of 14 participants dropped out of the control group at 3-month follow-up with no dropout in the intervention group (Figure 3), "

13b-i) Attrition diagram

Strongly recommended: An attrition diagram (e.g., proportion of participants still logging in or using the intervention/comparator in each group plotted over time, similar to a survival curve) or other figures or tables demonstrating usage/dose/engagement.

	1	2	3	4	5	
subitem not at all important	○	○	○	○	◉	essential

Rensa markering

Does your paper address subitem 13b-i?

Copy and paste relevant sections from the manuscript or cite the figure number if applicable (include quotes in quotation marks "like this" to indicate direct quotes from your manuscript), or elaborate on this item by providing additional information not in the ms, or briefly explain why the item is not applicable/relevant for your study

Please see Figure 3 for the relevant information.

14a) Dates defining the periods of recruitment and follow-up

Does your paper address CONSORT subitem 14a? *

Copy and paste relevant sections from the manuscript (include quotes in quotation marks "like this" to indicate direct quotes from your manuscript), or elaborate on this item by providing additional information not in the ms, or briefly explain why the item is not applicable/relevant for your study

"A parallel, open-label, 2-arm prospective, proof-of-concept pilot RCT with allocation ratio 1:1 was carried out in an outpatient clinical setting in the Department of Neurological Rehabilitation, University Hospital of Umeå, Sweden, from July 2020 to December 2021. "
"All participants received two outpatient visits at 3- and 12-months after stroke onset"

14a-i) Indicate if critical “secular events” fell into the study period

Indicate if critical “secular events” fell into the study period, e.g., significant changes in Internet resources available or “changes in computer hardware or Internet delivery resources”

	1	2	3	4	5	
subitem not at all important	○	◉	○	○	○	essential

Rensa markering

Does your paper address subitem 14a-i?

Copy and paste relevant sections from the manuscript (include quotes in quotation marks "like this" to indicate direct quotes from your manuscript), or elaborate on this item by providing additional information not in the ms, or briefly explain why the item is not applicable/relevant for your study

No such change has made during the study.
"In order to catch the minor but important changes on both daily activity and social participation over time, we added the Domain 8 in SIS (SIS-D8) [24] as another primary outcome to use in the future definitive RCT, since mRS covers mainly daily activity [15]. "

14b) Why the trial ended or was stopped (early)

Does your paper address CONSORT subitem 14b? *

Copy and paste relevant sections from the manuscript (include quotes in quotation marks "like this" to indicate direct quotes from your manuscript), or elaborate on this item by providing additional information not in the ms, or briefly explain why the item is not applicable/relevant for your study

It's not relevant in the current feasibility study

15) A table showing baseline demographic and clinical characteristics for each group
NPT: When applicable, a description of care providers (case volume, qualification, expertise, etc.) and centers (volume) in each group

Does your paper address CONSORT subitem 15? *

Copy and paste relevant sections from the manuscript (include quotes in quotation marks "like this" to indicate direct quotes from your manuscript), or elaborate on this item by providing additional information not in the ms, or briefly explain why the item is not applicable/relevant for your study

Please see Table 2 and relevant paragraphs

15-i) Report demographics associated with digital divide issues

In ehealth trials it is particularly important to report demographics associated with digital divide issues, such as age, education, gender, social-economic status, computer/Internet/ehealth literacy of the participants, if known.

	1	2	3	4	5	
subitem not at all important	○	○	○	○	◉	essential

Rensa markering

Does your paper address subitem 15-i? *

Copy and paste relevant sections from the manuscript (include quotes in quotation marks "like this" to indicate direct quotes from your manuscript), or elaborate on this item by providing additional information not in the ms, or briefly explain why the item is not applicable/relevant for your study

Please see Table 2 for the detail.

16) For each group, number of participants (denominator) included in each analysis and whether the analysis was by original assigned groups

16-i) Report multiple “denominators” and provide definitions

Report multiple “denominators” and provide definitions: Report N’s (and effect sizes) “across a range of study participation [and use] thresholds” [1], e.g., N exposed, N consented, N used more than x times, N used more than y weeks, N participants “used” the intervention/comparator at specific pre-defined time points of interest (in absolute and relative numbers per group). Always clearly define “use” of the intervention.

	1	2	3	4	5	
subitem not at all important	○	◉	○	○	○	essential

Rensa markering

Does your paper address subitem 16-i? *

Copy and paste relevant sections from the manuscript (include quotes in quotation marks "like this" to indicate direct quotes from your manuscript), or elaborate on this item by providing additional information not in the ms, or briefly explain why the item is not applicable/relevant for your study

It's not relevant in the current feasibility study

16-ii) Primary analysis should be intent-to-treat

Primary analysis should be intent-to-treat, secondary analyses could include comparing only “users”, with the appropriate caveats that this is no longer a randomized sample (see 18-i).

	1	2	3	4	5	
subitem not at all important	○	○	○	○	◉	essential

Rensa markering

Does your paper address subitem 16-ii?

Copy and paste relevant sections from the manuscript (include quotes in quotation marks "like this" to indicate direct quotes from your manuscript), or elaborate on this item by providing additional information not in the ms, or briefly explain why the item is not applicable/relevant for your study

"Patient Recruitment and Feasibility Assessments
One hundred patients were assessed for eligibility (Figure 3) from July 2020 to March 2021, to a high extent coinciding with the second wave of COVID-19 in Sweden. Twenty-eight participants gave written consent, which equated to a recruitment rate of 28%. Among the 72 patients who did not participate in the study (Figure 3), 50 patients never responded to the study invitation letter; 4 patients did not meet inclusion criteria; six patients declined without giving a reason; and one patient died. The remaining 11 patients (11%) declined participation due to various technical issues, such as no computer at home, no Internet, no BankID, and/or inability to use these technologies.
Four of 14 participants dropped out of the control group at 3-month follow-up with no dropout in the intervention group (Figure 3), resulting in a total trial completion rate of 86%, which was higher than the predefined cutoff (>60%) adherence to the study protocol (Table 1).
All 14 participants in the intervention group at 3-month follow-up and all 24 participants at 12-month follow-up used Rehabkompassen®, which gave 100% on the feasibility of the instrument. This was much better than the predefined cutoff (>60%) (Table 1). Satisfaction with the tool was reported among 79% of the patients and 100% of the physicians. Moreover, 75% of patients and both physicians would prefer to use the tool in the future

17a) For each primary and secondary outcome, results for each group, and the estimated effect size and its precision (such as 95% confidence interval)

Does your paper address CONSORT subitem 17a? *

Copy and paste relevant sections from the manuscript (include quotes in quotation marks "like this" to indicate direct quotes from your manuscript), or elaborate on this item by providing additional information not in the ms, or briefly explain why the item is not applicable/relevant for your study

It's not relevant in the current feasibility study

17a-i) Presentation of process outcomes such as metrics of use and intensity of use

In addition to primary/secondary (clinical) outcomes, the presentation of process outcomes such as metrics of use and intensity of use (dose, exposure) and their operational definitions is critical. This does not only refer to metrics of attrition (13-b) (often a binary variable), but also to more continuous exposure metrics such as "average session length". These must be accompanied by a technical description how a metric like a "session" is defined (e.g., timeout after idle time) [1] (report under item 6a).

	1	2	3	4	5	
subitem not at all important	○	◉	○	○	○	essential

Rensa markering

Does your paper address subitem 17a-i?

Copy and paste relevant sections from the manuscript (include quotes in quotation marks "like this" to indicate direct quotes from your manuscript), or elaborate on this item by providing additional information not in the ms, or briefly explain why the item is not applicable/relevant for your study

It's not relevant in the current study.
"All 14 participants in the intervention group at 3-month follow-up and all 24 participants at 12-month follow-up used Rehabkompassen®, which gave 100% on the feasibility of the instrument. "

17b) For binary outcomes, presentation of both absolute and relative effect sizes is recommended

Does your paper address CONSORT subitem 17b? *

Copy and paste relevant sections from the manuscript (include quotes in quotation marks "like this" to indicate direct quotes from your manuscript), or elaborate on this item by providing additional information not in the ms, or briefly explain why the item is not applicable/relevant for your study

"Although no statistically significant difference is expected to be found in this feasibility study, the differences on the primary and secondary outcomes on ordinal scale at the 12-month follow-up between intervention and control groups were tested using ordinal logistic regression."

18) Results of any other analyses performed, including subgroup analyses and adjusted analyses, distinguishing pre-specified from exploratory

Does your paper address CONSORT subitem 18? *

Copy and paste relevant sections from the manuscript (include quotes in quotation marks "like this" to indicate direct quotes from your manuscript), or elaborate on this item by providing additional information not in the ms, or briefly explain why the item is not applicable/relevant for your study

It's not relevant in the current feasibility study

18-i) Subgroup analysis of comparing only users

A subgroup analysis of comparing only users is not uncommon in ehealth trials, but if done, it must be stressed that this is a self-selected sample and no longer an unbiased sample from a randomized trial (see 16-iii).

	1	2	3	4	5	
subitem not at all important	(●)	( )	( )	( )	( )	essential

Rensa markering

Does your paper address subitem 18-i?

Copy and paste relevant sections from the manuscript (include quotes in quotation marks "like this" to indicate direct quotes from your manuscript), or elaborate on this item by providing additional information not in the ms, or briefly explain why the item is not applicable/relevant for your study

It's not relevant in the current feasibility study

19) All important harms or unintended effects in each group
(for specific guidance see CONSORT for harms)

Does your paper address CONSORT subitem 19? *

Copy and paste relevant sections from the manuscript (include quotes in quotation marks "like this" to indicate direct quotes from your manuscript), or elaborate on this item by providing additional information not in the ms, or briefly explain why the item is not applicable/relevant for your study

This has been addressed in the previous study (reference no.5 in the article)

19-i) Include privacy breaches, technical problems

Include privacy breaches, technical problems. This does not only include physical “harm” to participants, but also incidents such as perceived or real privacy breaches [1], technical problems, and other unexpected/unintended incidents. “Unintended effects” also includes unintended positive effects [2].

	1	2	3	4	5	
subitem not at all important	○	◉	○	○	○	essential

Rensa markering

Does your paper address subitem 19-i?

Copy and paste relevant sections from the manuscript (include quotes in quotation marks "like this" to indicate direct quotes from your manuscript), or elaborate on this item by providing additional information not in the ms, or briefly explain why the item is not applicable/relevant for your study

This has been addressed in the previous study (reference no.5 in the article)

19-ii) Include qualitative feedback from participants or observations from staff/researchers

Include qualitative feedback from participants or observations from staff/researchers, if available, on strengths and shortcomings of the application, especially if they point to unintended/unexpected effects or uses. This includes (if available) reasons for why people did or did not use the application as intended by the developers.

	1	2	3	4	5	
subitem not at all important	○	○	○	○	●	essential

Rensa markering

Does your paper address subitem 19-ii?

Copy and paste relevant sections from the manuscript (include quotes in quotation marks "like this" to indicate direct quotes from your manuscript), or elaborate on this item by providing additional information not in the ms, or briefly explain why the item is not applicable/relevant for your study

"Post-visit Assessments of Satisfaction with the Rehabkompassen®
Acceptability was assessed in terms of the patient-participants' satisfaction with Rehabkompassen®. After the 3- and 12-month follow-up visits, all patient-participants in both intervention and control groups answered a satisfaction questionnaire through 1177.se. "
"In the end of the 3-month follow-ups, two physicians involved in the study answered a questionnaire with 5 questions regarding different aspects of utility for providing feedback on perceived feasibility and satisfaction of the instrument in clinical practice."

DISCUSSION

22) Interpretation consistent with results, balancing benefits and harms, and considering other relevant evidence

NPT: In addition, take into account the choice of the comparator, lack of or partial blinding, and unequal expertise of care providers or centers in each group

22-i) Restate study questions and summarize the answers suggested by the data, starting with primary outcomes and process outcomes (use)

Restate study questions and summarize the answers suggested by the data, starting with primary outcomes and process outcomes (use).

	1	2	3	4	5	
subitem not at all important	○	○	○	○	◉	essential

Rensa markering

Does your paper address subitem 22-i? *

Copy and paste relevant sections from the manuscript (include quotes in quotation marks "like this" to indicate direct quotes from your manuscript), or elaborate on this item by providing additional information not in the ms, or briefly explain why the item is not applicable/relevant for your study

"This randomized clinical feasibility study investigated the feasibility and acceptability of conducting a definitive trial of evaluating Rehabkompassen® as a digital follow-up tool among persons with stroke in an outpatient clinic setting. The overall recruitment rate was 28%. Retention in the trial was 86% at 12-month follow-up, which indicated high adherence to study protocol. ...."

22-ii) Highlight unanswered new questions, suggest future research

Highlight unanswered new questions, suggest future research.

	1	2	3	4	5	
subitem not at all important	○	○	○	○	◉	essential

Rensa markering

Does your paper address subitem 22-ii?

Copy and paste relevant sections from the manuscript (include quotes in quotation marks "like this" to indicate direct quotes from your manuscript), or elaborate on this item by providing additional information not in the ms, or briefly explain why the item is not applicable/relevant for your study

"At this stage, the results remain hardly to be generalized due to its limited sample size (two physicians, 14 patients in contro- and intervention groups respectively) in this pilot study, a further definitive RCT is thus needed"

20) Trial limitations, addressing sources of potential bias, imprecision, and, if relevant, multiplicity of analyses

20-i) Typical limitations in ehealth trials

Typical limitations in ehealth trials: Participants in ehealth trials are rarely blinded. Ehealth trials often look at a multiplicity of outcomes, increasing risk for a Type I error. Discuss biases due to non-use of the intervention/usability issues, biases through informed consent procedures, unexpected events.

	1	2	3	4	5	
subitem not at all important	○	○	○	○	◉	essential

Rensa markering

Does your paper address subitem 20-i? *

Copy and paste relevant sections from the manuscript (include quotes in quotation marks "like this" to indicate direct quotes from your manuscript), or elaborate on this item by providing additional information not in the ms, or briefly explain why the item is not applicable/relevant for your study

"Although the current feasibility study provides important information and necessary amendments for the future definitive RCT study, there are a couple of limitations of this feasibility study. Since this feasibility study was carried out in only one outpatient clinic, we cannot generalize the results directly into different participating clinics with various clinical routines in the future multicenter RCT. It remains a challenge to fit the Rehabkompassen® tool within various existing clinical routines despite the great rates of feasibility and acceptability demonstrated in the current study. Furthermore, this feasibility study was performed by an experienced clinical research team, which is crucial for reaching a high-quality study. Therefore, it is very important that knowledge transfer, timely trouble-shooting, and problem-solving by the experienced research team be available during the future definitive RCT. ."

21) Generalisability (external validity, applicability) of the trial findings

NPT: External validity of the trial findings according to the intervention, comparators, patients, and care providers or centers involved in the trial

21-i) Generalizability to other populations

Generalizability to other populations: In particular, discuss generalizability to a general Internet population, outside of a RCT setting, and general patient population, including applicability of the study results for other organizations

	1	2	3	4	5	
subitem not at all important	○	○	○	○	●	essential

Rensa markering

Does your paper address subitem 21-i?

Copy and paste relevant sections from the manuscript (include quotes in quotation marks "like this" to indicate direct quotes from your manuscript), or elaborate on this item by providing additional information not in the ms, or briefly explain why the item is not applicable/relevant for your study

"At this stage, the results remain hardly to be generalized due to its limited sample size (two physicians, 14 patients in contro- and intervention groups respectively) in this pilot study, a further definitive RCT is thus needed"

21-ii) Discuss if there were elements in the RCT that would be different in a routine application setting

Discuss if there were elements in the RCT that would be different in a routine application setting (e.g., prompts/reminders, more human involvement, training sessions or other co-interventions) and what impact the omission of these elements could have on use, adoption, or outcomes if the intervention is applied outside of a RCT setting.

	1	2	3	4	5	
subitem not at all important	○	○	●	○	○	essential

Rensa markering

Does your paper address subitem 21-ii?

Copy and paste relevant sections from the manuscript (include quotes in quotation marks "like this" to indicate direct quotes from your manuscript), or elaborate on this item by providing additional information not in the ms, or briefly explain why the item is not applicable/relevant for your study

This has been addressed in the previous study (reference no.5 in the article)

OTHER INFORMATION

23) Registration number and name of trial registry

Does your paper address CONSORT subitem 23? *

Copy and paste relevant sections from the manuscript (include quotes in quotation marks "like this" to indicate direct quotes from your manuscript), or elaborate on this item by providing additional information not in the ms, or briefly explain why the item is not applicable/relevant for your study

ClinicalTrials.gov Identifier: NCT04915027

24) Where the full trial protocol can be accessed, if available

Does your paper address CONSORT subitem 24? *

Cite a Multimedia Appendix, other reference, or copy and paste relevant sections from the manuscript (include quotes in quotation marks "like this" to indicate direct quotes from your manuscript), or elaborate on this item by providing additional information not in the ms, or briefly explain why the item is not applicable/relevant for your study

ClinicalTrials.gov Identifier: NCT04915027

25) Sources of funding and other support (such as supply of drugs), role of funders

Does your paper address CONSORT subitem 25? *

Copy and paste relevant sections from the manuscript (include quotes in quotation marks "like this" to indicate direct quotes from your manuscript), or elaborate on this item by providing additional information not in the ms, or briefly explain why the item is not applicable/relevant for your study

"Funding: This study was supported by VINNOVA Medtech4Health (2019-01389), Västerbotten County Council and Umeå University (ALF Foundation), the Swedish Stroke Foundation (Stroke Riksförbundet), the Northern Swedish Stroke Fund (Strokeforskning I Norrland Insamlingsstiftelse), Heart-Lung foundation (2020676), and Forte (2020-00136)."

X27) Conflicts of Interest (not a CONSORT item)

X27-i) State the relation of the study team towards the system being evaluated

In addition to the usual declaration of interests (financial or otherwise), also state the relation of the study team towards the system being evaluated, i.e., state if the authors/evaluators are distinct from or identical with the developers/sponsors of the intervention.

	1	2	3	4	5	
subitem not at all important	○	○	○	○	◉	essential

Rensa markering

Does your paper address subitem X27-i?

Copy and paste relevant sections from the manuscript (include quotes in quotation marks "like this" to indicate direct quotes from your manuscript), or elaborate on this item by providing additional information not in the ms, or briefly explain why the item is not applicable/relevant for your study

Nothing to declare.
"Conflicts of Interest, None declared"

About the CONSORT EHEALTH checklist

As a result of using this checklist, did you make changes in your manuscript? *

○ yes, major changes

○ yes, minor changes

◉ no

What were the most important changes you made as a result of using this checklist?

Ditt svar

How much time did you spend on going through the checklist INCLUDING making changes in your manuscript *

Several hours!! It's really not necessary!!!"

As a result of using this checklist, do you think your manuscript has improved? *

- ( ) yes
- (•) no
- ( ) Övrigt:

Would you like to become involved in the CONSORT EHEALTH group?

This would involve for example becoming involved in participating in a workshop and writing an "Explanation and Elaboration" document

- ( ) yes
- (•) no
- ( ) Övrigt:

Rensa markering

Any other comments or questions on CONSORT EHEALTH

Ditt svar

STOP - Save this form as PDF before you click submit
To generate a record that you filled in this form, we recommend to generate a PDF of this page (on a Mac, simply select "print" and then select "print as PDF") before you submit it.

When you submit your (revised) paper to JMIR, please upload the PDF as supplementary file.

Don't worry if some text in the textboxes is cut off, as we still have the complete information in our database. Thank you!

Final step: Click submit !
Click submit so we have your answers in our database!

Skicka Rensa formuläret

Skicka aldrig lösenord med Google Formulär

Det här innehållet har varken skapats eller godkänts av Google. Anmäl otillåten användning - Användarvillkor - Integritetspolicy

Google Formulär
